# Epithelial-to-mesenchymal transition and NF-kB pathways are promoted by a mutant form of DDB2, unable to bind PCNA, in UV-damaged human cells

**DOI:** 10.1186/s12885-024-12368-6

**Published:** 2024-05-21

**Authors:** Paola Perucca, Elisabetta Bassi, Martina Vetro, Anna Tricarico, Ennio Prosperi, Lucia Anna Stivala, Ornella Cazzalini

**Affiliations:** 1 Dipartimento di Medicina molecolare, Unità di Immunologia e Patologia generale, Università degli Studi di Pavia, Pavia, Italy; 2 Istituto di Genetica Molecolare (IGM) del CNR, Pavia, Italy

**Keywords:** DNA-Damaged binding protein 2, Epithelial-mesenchymal transition, Invasion, p-EMT, EMT-TFs

## Abstract

**Background:**

DNA-Damaged Binding protein 2 (DDB2) is a protein involved in the early step of Nucleotide Excision Repair. Recently, it has been reported that DDB2 is involved in epithelial-to-mesenchymal transition (EMT), key process in tumour invasiveness and metastasis formation. However, its role is not completely known.

**Methods:**

Boyden chamber and cell adhesion assays, and ICELLigence analysis were performed to detect HEK293 adhesion and invasion. Western blotting and gelatine zymography techniques were employed to assess the EMT protein levels and MMP enzymatic activity. Immunofluorescence analysis and pull-down assays facilitated the detection of NF-kB sub-cellular localization and interaction.

**Results:**

We have previously demonstrated that the loss of DDB2-PCNA binding favours genome instability, and increases cell proliferation and motility. Here, we have investigated the phenotypic and molecular EMT-like changes after UV DNA damage, in HEK293 clones stably expressing DDB2^Wt^ protein or a mutant form unable to interact with PCNA (DDB2^PCNA−^), as well as in HeLa cells transiently expressing the same DDB2 constructs. Cells expressing DDB2^PCNA−^ showed morphological modifications along with a reduced expression of E-cadherin, an increased activity of MMP-9 and an improved ability to migrate, in concomitance with a significant upregulation of EMT-associated Transcription Factors (TFs), whose expression has been reported to favour tumour invasion. We observed a higher expression of c-Myc oncogene, NF-kB, both regulating cell proliferation and metastatic process, as well as ZEB1, a TF significantly associated with tumorigenic potential and cell migratory ability. Interestingly, a novel interaction of DDB2 with NF-kB was detected and found to be increased in cells expressing the DDB2^PCNA−^, suggesting a direct modulation of NF-kB by DDB2.

**Conclusion:**

These results highlight the role of DDB2-PCNA interaction in counteracting EMT since DDB2^PCNA−^ protein induces in HEK293 transformed cells a gain of function contributing to the acquisition of a more aggressive phenotype.

**Supplementary Information:**

The online version contains supplementary material available at 10.1186/s12885-024-12368-6.

## Introduction

DDB2 protein is involved in Nucleotide Excision Repair as a sensor of DNA damages caused by UV irradiation [1]. However, recently some papers reported new roles of this protein in the development and progression of various types of cancers [reviewed in 2]. Cancer is a multifaceted disease showing its aggressiveness through different processes, among these, the epithelial-mesenchymal transition (EMT). Very recently, it has been highlighted that multiple transitional states (E/M phenotypes) expressing mixed epithelial and mesenchymal genes, also referred as partial EMT (p-EMT) are included in many aggressive human cancers, but the molecular mechanisms controlling this transition are still unknown [3–5].

DDB2 has been shown to target multiple signalling molecules playing a role in EMT process. In fact, it appears able to inhibit the EMT process by regulating HIF1α expression [6], or through transcriptional repression of SNAIL1, ZEB1 and VEGF in colon, oral and neck tumours [7, 8], and to repress EMT in pancreatic ductal adenocarcinoma cells sensitizing cancer cells to chemotherapy [9]. In ovarian and breast cancer, DDB2 up-regulates IkBα, an inhibitor of NF-kB, and interacts with TGF-β1 signalling pathways, inhibiting cancer cell EMT. It is known that NF-kB protein plays an important role in EMT activation, invasion and metastasis processes as well as in tumour cell proliferation; for these reasons, NF-kB is considered one of the most important molecules linking chronic inflammation to cancer [10]. Moreover, NF-kB increases transcription of oncogenic driver genes such as c-Myc [11] and it also blocks the apoptotic events favouring cell survival [12]. NF-kB also promotes ZEB1 expression; ZEB1 is one of the EMT-Transcription Factors (EMT-TFs) which, by downregulating E-Cadherin expression, activates the EMT process. Recently, ZEB1 has been indicated as a factor that induces cancer proliferation and drug resistance favouring the growth and viability of cancer cells [13]. Thus, high expression level of ZEB1 is correlated with a poor outcome in cancer patients [14].

These lines of evidence indicate that DDB2 may act as EMT suppressor. In contrast, other results have shown that DDB2 determines the degradation of PAQR3, thus increasing cell migration and invasion of gastric cancer cells [15]. In addition, DDB2 may be involved in cell adhesion by modulating membrane mechanical properties in mammary cancer cells [16] and promoting proliferation in breast cancer cells [17]. Therefore, the role of DDB2 in EMT control remains to be clarified.

We have previously shown that a mutant form of DDB2 (DDB2^PCNA−^), unable to interact with PCNA [18] increases human embryonic kidney cell (HEK293) proliferation and motility after UV irradiation [19]. Because of this, in this work we focused on the occurrence of further phenotypic or molecular alterations underlying the EMT process in cells expressing constitutively this mutant form of DDB2. In particular, we have investigated morphological changes and motility, as well as specific markers of EMT, such as E-Cadherin and Vimentin, and matrix-metalloproteinases (MMP-2 and MMP-9). In addition, we have investigated the molecular mechanism by which DDB2 might influence EMT, focusing on the expression level of specific transcription factors in our cell model systems. In addition, anticancer drug-sensitivity of HEK293 clones was also evaluated, together with the level and activity of Hypoxanthine guanine phosphoribosyltransferase (HPRT).

Our results show that DDB2^PCNA−^ stimulates the appearance of typical EMT features in HEK293, also confirmed in HeLa cells, indicating that this mutation induces a gain of function contributing to the acquisition of a mesenchymal phenotype in HEK293 transformed cells.

## Materials and methods

### Cell lines and transfection

HEK293 (Human Embryonic Kidney) cell line was purchased from the ECACC (code 85120602) (CLS Cat# 300192/p777_HEK293, RRID: CVCL_0045). The cell line was cultured in Dulbecco’s modified Eagle’s medium (DMEM, Sigma) supplemented with 10% fetal bovine serum (Life Technologies-Gibco), 2mM L-glutamine (Life Technologies-Gibco), 100 U/ml penicillin, 100 µg/ml streptomycin, in a 5% CO_2_ atmosphere.

Cells were stably transfected with DDB2^Wt^ or DDB2^PCNA−^, as previously reported [18]. Endogenous DDB2 expression level in HEK293 cells was assessed in our previous published paper [20]. Parental (referred to as Control) and stably transfected HEK293 clones were exposed to UV-C irradiation (10 J/m^2^, 20 s) in all the experiments, in order to induce DNA damage and activate a DDB2-dependent DNA damage response.

HeLa endocervical carcinoma cells (ATCC) were kindly provided by A.I. Scovassi (IGM-CNR Pavia) were grown in Dulbecco modified Eagle medium (DMEM, Sigma) supplemented with 10% fetal bovine serum (FBS, Gibco BRL), 4 mM L-glutamine (Gibco BRL), 100 U/ml penicillin, 100 µg/ml streptomycin in a 5% CO_2_ atmosphere. Cells seeded on coverslips were transiently transfected 24 h later with Effectene transfection reagent (Qiagen) (about 70% confluence) using the same constructs above described, and irradiation was usually performed 24 h after transfection.

### Morphological analysis

Control and stably transfected HEK293 were seeded (1 × 10^5^ cells) on coverslips, irradiated and analysed after 3 days with an inverted light microscope equipped with a Canon A590 IS camera (Tokyo, JP). Cell length analysis was performed using the public software ImageJ (https://imagej.nih.gov/ij/ RRID: SCR_003070).

### Western blot analysis and gelatin zymography

Control and stably transfected HEK293 clones expressing DDB2^Wt^ or DDB2^PCNA−^ protein were exposed to UV-C irradiation (10 J/m^2^). After different recovery times (4, 8, 24, 48, 72, 96 and 168 h) from UV-induced DNA damage, pelleted cells were collected. For blot analysis, the cells were directly lysed in SDS sample buffer (65 mM Tris-HCl pH 7.5, 1% SDS, 30 mM dithiothreitol (DTT), 10% glycerol, 0.02% Bromophenol Blue).

Proteins were resolved by SDS-PAGE (gel 10%) and membranes probed with primary antibodies anti-E-Cadherin 1:1000 (GeneTex Cat# GTX100443, RRID: AB_10729586), anti-Vimentin 1:1000 (Santa Cruz Biotechnology Cat# sc-6260, RRID: AB_628437), anti-ZEB1 (H3) 1:1000 (Santa Cruz Biotechnology Cat# sc-515797, RRID: AB_2934316), anti-NFkappaB p65 (F-6) 1:500 (Santa Cruz Biotechnology Cat# sc-8008, RRID: AB_628017), Anti-SNAI1 (E-10) 1:500 (Santa Cruz Biotechnology Cat# sc-393172, RRID: AB_2938534), anti-c-Myc (9E10) 1:750 (Santa Cruz Biotechnology Cat# sc-40, RRID: AB_627268), anti-PCNA (PC10) 1:1000 (Agilent Cat# M0879, RRID: AB_2160651), MCM2 (BM28) 1:1000 (BD Biosciences Cat# 610700, RRID: AB_2141952); anti-Actin 1:1000 (Sigma-Aldrich Cat# A4700, RRID: AB_476730), and anti-HPRT (F-1) 1:500 (Santa Cruz Biotechnology Cat# sc-376938, RRID: AB_2938532), then incubated with anti-rabbit HRP 1:10000 (Sigma-Aldrich Cat# A9169, RRID: AB_258434) and anti-mouse HRP 1:10000 (Sigma-Aldrich Cat# A9044, RRID: AB_258431) secondary antibodies. Membranes were cut in different strips to allow detection of the relevant proteins in the same run. The uncropped original blots are shown in the supplementary materials. The signal was revealed using enhanced chemiluminescence by Azure c600 Gel Imaging System (Azure Biosystem). The quantification of the bands was done compared to the loading control (actin) on the same blot, by the public software ImageJ (https://imagej.nih.gov/ij/ RRID: SCR_003070).

The culture media from each sample were used to test MMP-2 and MMP-9 activity by gelatin zymography, as previously reported [21]. Gel images were acquired by a Canon Canoscan 9950 F and densitometric analysis was performed by the public software as above. All the experiments were carried out at least 3 times.

### Pull-down and western blot analysis

Control and transfected cells were lysed 7d after UV-C in hypotonic buffer containing 10 mM Tris-HCl (pH 7.4), 2.5 mM MgCl_2_, 1 mM PMSF, 0.5% Nonidet NP-40, Imidazole 10 mM and a mixture of protease and phosphatase inhibitor cocktails (Sigma). After 10 min on ice, the cells were sonicated, quantified with Bradford assay and the samples for the input were collected.

For pull-down experiments, equal amount (1,5 mg/ml of proteins) of each sample was incubated with Ni-NTA resin (Qiagen). The reactions were performed over night at 4 °C under constant agitation.

The samples were then centrifuged at 14,000 g (30 min, 4 °C), washed with ice-cold 50 mM Tris-HCl (pH 7.4), and eluted in SDS sample buffer and resolved by SDS-PAGE. Western blot and densitometric analysis were performed as reported above. The primary antibodies used are: p48-DDB2 (Rockland Cat# 100-401-A10, RRID: AB_2276988) anti-NF-kB p65 (F-6) 1:500 (Santa Cruz Biotechnology Cat# sc-8008, RRID: AB_628017). The HRP-conjugated secondary antibodies are: anti-rabbit HRP 1:10000 (Sigma-Aldrich Cat# A9169,RRID: AB_258434) and anti-mouse HRP 1:10000 (Sigma-Aldrich Cat# A9044, RRID: AB_258431).

### Immunofluorescence analysis

Control and stably transfected HEK293 clones expressing DDB2^Wt^ or DDB2^PCNA−^ protein were seeded on coverslips (22 × 22 mm) and exposed to UV-C irradiation (10 J/m^2^). Seven days after UV damage, the cells were washed twice with PBS buffer, fixed in 2% formaldehyde for 5 min at r.t., and then post-fixed in 70% ethanol. After rehydration in PBS, blocking of unspecific staining was performed in PBT solution (PBS, 0.2% Tween 20). Immunostaining of NF-kB was performed by incubation with the antibody: anti-NF-kB p65 (F-6) 1:500 (Santa Cruz Biotechnology Cat# sc-8008, RRID: AB_628017). After washing in PBT, the coverslips were incubated for 30 min with anti-mouse conjugated with Alexa 488 1:100 (Molecular Probes Cat# A-21202, RRID: AB_141607). Then, cells were stained with Hoechst 33258 dye (0.5 mg/ml) for 5 min at r.t. and washed in PBS.

HeLa cells seeded on coverslips were transfected as described above. After 24 h, the cells were irradiated and re-incubated in complete medium for 3 days. The cells on coverslips were then washed in PBS, fixed in paraformaldehyde 2% and permeabilized in ethanol 70%. After the samples were blocked in PBST buffer (PBS, 0.2% Tween 20) containing 1% bovine serum albumin (BSA), and then incubated for 1 h with specific antibodies: p48-DDB2 (Rockland Cat# 100-401-A10, RRID: AB_2276988) anti-Vimentin 1:1000 (Santa Cruz Biotechnology Cat# sc-6260, RRID: AB_628437) all diluted in PBST buffer/BSA. After washing, each reaction was followed by incubation for 30 min with anti-rabbit conjugated (Molecular Probes Cat# A-21206 (also A21206), RRID: AB_2535792) and with anti-mouse conjugated (Thermo Fisher Scientific Cat# 35510, RRID: AB_1185569). After immunoreactions, the cells were incubated with Hoechst 33258 dye (0.5 µg/ml) for 5 min at RT and washed in PBS. The slides were mounted in Mowiol (Calbiochem) containing 0.25% 1,4- diazabicyclo-octane (Aldrich) as antifading agent. Images of fixed cells were taken with Nikon Eclipse E400 fluorescence microscope with a 100X objective (NA 1.25). For each condition, the experiment was repeated at least 3 times. Fluorescence photographs of representative samples were taken with Canon Power Shot A590IS digital camera.

### Boyden chamber assay

The Boyden chamber (Neuroprobe, Gaithersburg, MD) was assembled by inserting collagen type 1 (100 µg/ml, from calf skin, Sigma-Aldrich) or Matrigel (BD MatrigelTM) coated filter. For each cell line, 10^5^ cells were diluted in 50 µl of medium without FBS and loaded in each well. Serum-free medium or medium containing 10% FBS was added to the bottom wells as a negative control or chemoattractant, respectively. Cell migration was evaluated by counting the migrated cells under a digital microscope Nikon Eclipse 80i with a camera Nikon Digital Sight DS-Fi1 [21].

### Collagen type I adhesion assay

96 well-plates were coated with 50 µl/well of collagen type I diluted in PBS (10 µg/ml), incubated overnight at 4 °C and then conditioned with 150 µl/well of 1% BSA in PBS for 30 min at r.t. to block non-specific sites. Irradiated HEK293 cells were seeded in 96 well-plate, and incubated for 30 min at 37 °C before being fixed and stained with Crystal violet. The cells were observed employing a Leica inverted light microscope equipped with a Canon A590 IS camera (Tokyo, JP). The percentage of adherent cells was calculated from the maximal adhesion rate [16].

### iCelligence assay

The iCELLigence System (ACEA Biosciences) is a microelectronic biosensor system for cell-based assays, providing dynamic, real-time cellular analysis for different tests, and among these, cell adhesion. The iCELLigence System consists of two components: the iCELLigence Control Unit and the iCELLigence Instrument with two integrated plate cradles for measuring cell responses in parallel.

The presence of cells on top of the electrodes will affect the local ionic environment at the electrode/solution interface, leading to an increase in the electrode impedance. An increase in the number of cells attached on the electrodes leads to an increase in electrode impedance [22].

HEK293 control line and both stable clones expressing DDB2^Wt^ or DDB2^PCNA−^ protein were exposed to UV-C irradiation (10 J/m^2^) and immediately harvested and counted. 10^4^ cells diluted in 400 µl of medium were loaded in each well. The adhesion was monitored for 10 h.

### Evaluation of 6-thioguanine (6-TG) cytotoxicity by MTT assay

Cells were seeded in 96-well tissue culture plates at the concentration of 2.5 × 10^4^ cells/well, treated for 24 h with 6-thioguanine (6-TG) at different concentrations of 1, 2.5, 5, 7 and 10 µM and cell toxicity was determined by the 3-[4,5-dimethylthiazolil-2yl]-2,5-diphenyl-tetrazoliumbromide colorimetric assay (MTT).

### Clonogenic efficiency after 6-thioguanine (6-TG) or cisplatin treatments

Control and stably transfected HEK293 cells, untreated or UV-C irradiated, were harvested, resuspended in fresh medium containing 6-TG and seeded in culture dishes. The concentration of 6-TG (2.5 µM) has been chosen after MTT assay.

For the cisplatin treatment, control and stable transfected HEK293 cells, were harvested, resuspended in fresh medium containing cisplatin 25 or 50 µM and seeded in culture dishes.

The cells were incubated for 10 d to allow colony formation. After fixation in ethanol, the colonies were stained with 0.1% crystal violet and counted by visual scoring. Clonogenic efficiency was evaluated as reported by Perucca et al. [19].

### Statistical analysis

Results are expressed as mean ± standard deviation. Statistical significance was calculated using the one-way Anova analyses. The Tukey’s Multiple Comparison test was used to compare means from several experimental groups. Data are means of at least 3 independent experiments.

## Results

### Morphological changes in HEK293 DDB2^PCNA−^ cells after UV-C irradiation

**Fig. 1:**
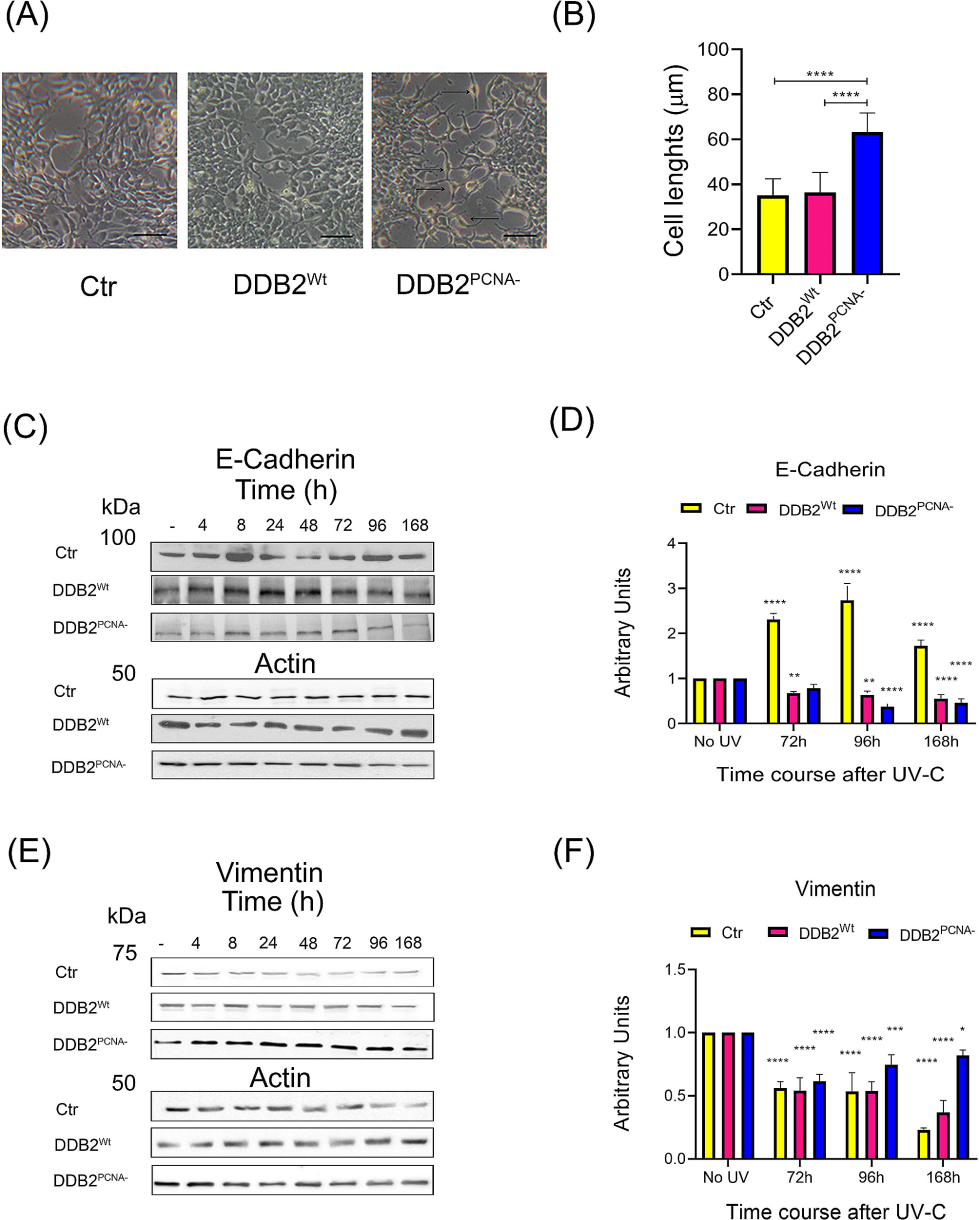
Epithelial-to-mesenchymal transition (EMT) analysis in UV-irradiated cells. Representative images of morphological phenotypes of UV-irradiated control (Ctr), DDB2^Wt^ or DDB2^PCNA−^ clones and the respective size cell measurements (**A** and **B**); arrows indicate a more elongated shape visible in the DDB2^PCNA−^ clone. Scale bar = 50 μm. E-Cadherin levels are reduced in HEK293 stably transfected with DDB2^PCNA−^ construct. Western blot analysis related to expression levels of E-Cadherin (**C**) and Vimentin (**E**). Cells were collected at different times after UV-C irradiation (10 J/m^2^), as described in Materials and Methods. Protein levels were normalized to actin values through densitometric analysis (**D** and **F**). Mean values (± S.D.) are from at least 3 independent experiments. For all samples, statistical significance is *versus* the respective unirradiated clones. Statistical significance was calculated using the one-way ANOVA with Tukey’s multiple comparison. *p < 0.05, **p < 0.01, ***p < 0.0001 and ****p < 0.00001. The complete time-course is shown in the Fig. S2. The uncropped original blots are shown in the supplementary file WB

In irradiated HEK293 cells expressing DDB2 exogenous protein, we performed a morphological analysis by microscopical analysis in order to investigate a possible activation of epithelial-to-mesenchymal transition (EMT). In fact, EMT has been associated with a process of conversion from a cuboidal epithelial structure into an elongated mesenchymal shape. These morphological modifications seem to be more evident in cells expressing DDB2^PCNA−^ (Fig. 1A), whose shape appears to be more elongated and thinner, compared to both irradiated DDB2 wild-type and control cells and the corresponding unirradiated cells (Fig. S1); measuring cell size in the field of lower cell density (Fig. 1B), irradiated DDB2^PCNA−^ cells show a length of 63 μm than that of about 34 μm of control and Wt clones after UV (*p* < 0.0001). These observations suggest an intrinsic plasticity of the epithelial phenotype in cells expressing DDB2 mutant protein resulting in these different dimensions.

### UV-induced DNA damage affects E-Cadherin and Vimentin levels in cells expressing DDB2^PCNA−^ protein

Control or stably expressing DDB2^Wt^ or DDB2^PCNA−^ HEK293 cells were irradiated and harvested at different times, as indicated in Fig. 1. The analysis of the E-Cadherin bands showed that the highest levels are found in the control irradiated cells, starting from 72 to 168 h after UV-C irradiation; in contrast, the DDB2^PCNA−^ clone showed a reduction of the protein levels, in particular at 96 and 168 h (Fig. 1C and D).

The results related to Vimentin (Fig. 1E and F) demonstrated their significative reduction in all samples after UV-exposure, as compared to unirradiated cell clones. Nevertheless, DDB2^PCNA−^ cells express the highest value of the protein, even though its expression did not significantly change compared to the other times, indicating that the trend remained stable over time. In contrast, in the other two cell clones, the reduction in Vimentin is evident starting from 72 h and it was more marked at 168 h (Fig. 1F**)**, as compared to the respective unirradiated samples. No significant changes were observed at the shorter times (from 4 to 48 h), as shown in the Fig. S2.

In order to confirm these results in another cell type, Vimentin expression was also analyzed in HeLa cells transiently transfected with DDB2^Wt^ or DDB2^PCNA−^ constructs. The results obtained by immunofluorescence analysis performed 3 days after UVC irradiation, are shown in Figure S3. In HeLa cells expressing DDB2^PCNA−^, Vimentin was clearly increased with respect to HeLa DDB2^Wt^ cells in which the protein was weakly detectable.

All together, these results suggest a possible p-EMT activation.

### HEK293 expressing DDB2^PCNA−^ protein modifies the adhesion ability after UV irradiation

**Fig. 2:**
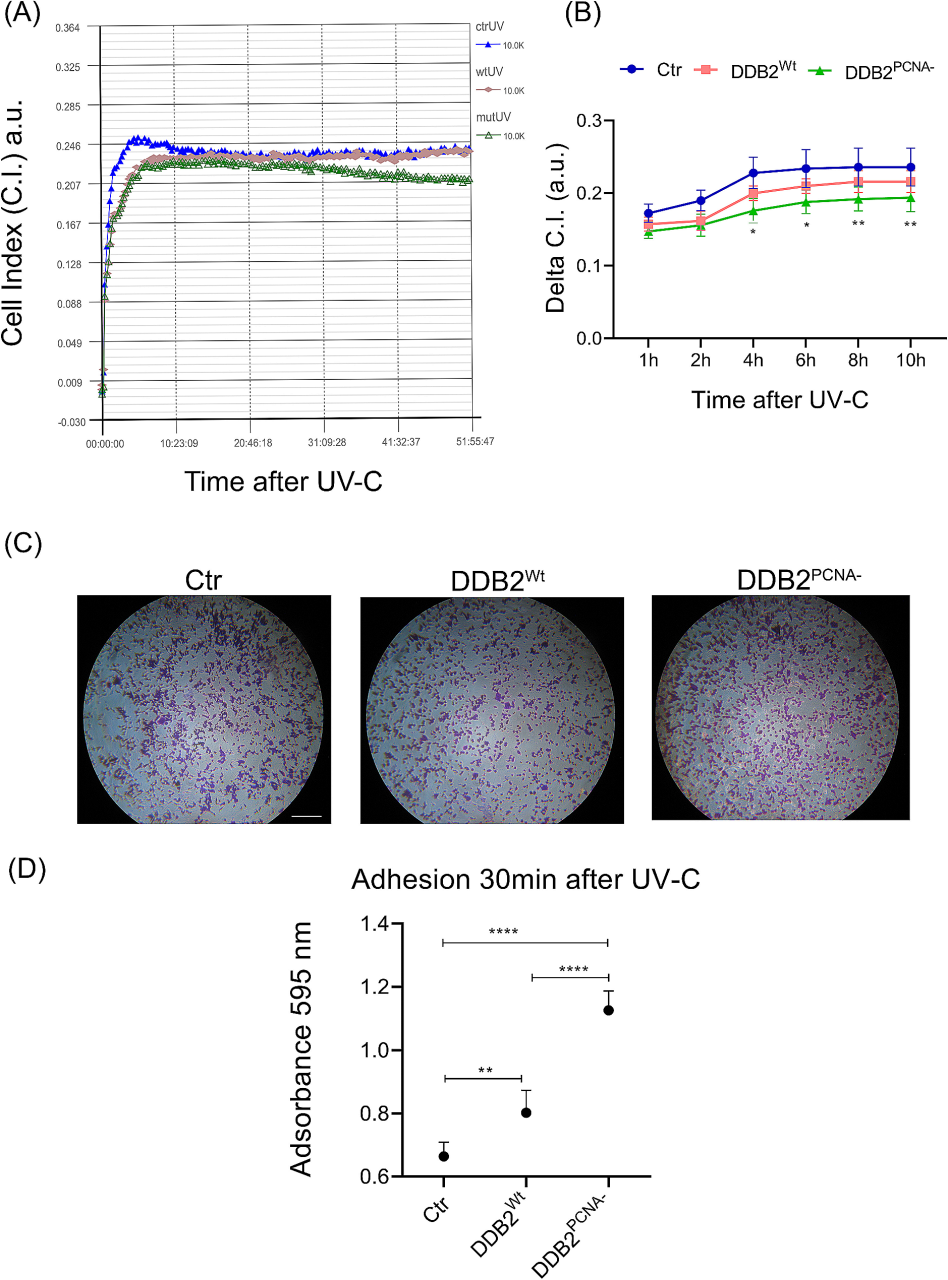
Cell adhesion ability in UV-irradiated HEK293 stable clones. (**A**) Representative sensorgram of irradiated cells. In (**B**) the results from ICELLigence analysis are shown. After UV-C irradiation, the cells are seeded on the provided supports and data are collected at different time, as described in Material and Methods section. Cell Index (C.I.) Quantification of delta cell index (dC.I.) obtained from RTCA until 10 h after UV-C irradiation. dC.I. is expressed as the CI variation at the endpoint with regard to the value obtained after seeding. In (**C**), cells irradiated, harvested and seeded were incubated 30 min at 37 °C. Then, cells were fixed, stained with Crystal Violet and photographed (Canon A590 IS). Scale bar = 500 μm. Then, cells are solubilized (see Materials and Method section) and the samples were quantified by spectrophotometer and results reported in (**D**). Data are the mean ± S.D. from at least three independent experiments. Statistical significance was calculated using the one-way ANOVA with Tukey’s multiple comparison.*p < 0.05, **p < 0.01, ****p < 0.0001

The adhesion assay was performed using iCELLigence real-time cell analysis (RTCA) and the data was collected at different time after UV irradiation and seeding. Mutant cells showed a delay in the adhesion (Fig. 2A) of cells on the iCELLigence support, which is not coated with adhesion substrates. The analysis reported in Fig. 2B shows that there is a different adhesion kinetics, which is significant starting from 4 h after UV damage. This result supports the idea that these cells have acquired a major ability to move than the control and DDB2^Wt^ cells.

To further investigate the influence of DDB2^PCNA−^ protein on cellular adhesion, we also performed experiments by seeding each cell clones on collagen I-coated 96-well plates. In Fig. 2C some representative images in which many cells, stained with Crystal violet, adhere to the well surface. To reliably quantify the results, we performed a spectrophotometric analysis, and data are reported in Fig. 2D. The mutant clone reveals a statistically significant (*p* < 0.0001) major ability to adhere to ECM than both control cells and DDB2^Wt^ clone, suggesting an enhanced capacity to stick on the dishes after UV-induced damage.

### Metalloproteinase 9, but not 2, increases the activity in the UV-irradiated DDB2^PCNA−^clone

**Fig. 3:**
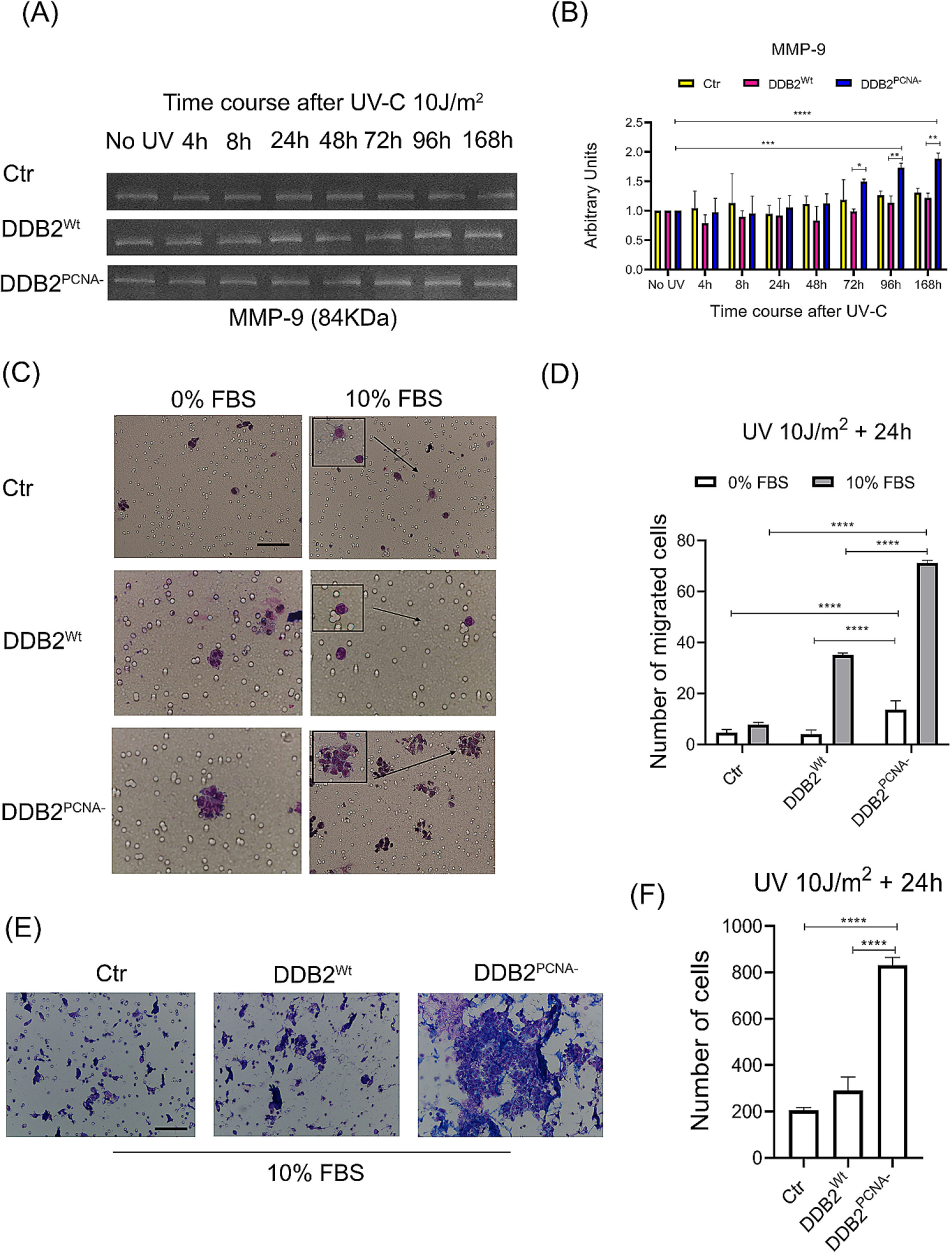
Cell migration and invasion capacities in UV-irradiated HEK293 stable clones. The loss of DDB2-PCNA interaction favours cell migration. Representative images of MMP-9 analysis performed in the three different clones by zymography are shown (**A**). Results from densitometric analysis are reported in (**B**). Data are mean ± SD from at least three independent experiments. Cell migration or invasion were evaluated using the Boyden chamber and filters treated with collagen type I (**C**) or Matrigel (**E**). Serum-free medium (0% FBS) and medium containing 10% FBS were applied to the bottom wells as negative control or chemoattractant, respectively. Data obtained by counting the migrated cells (**D**) or invading cells (**F**) are the mean ± SD from at least three independent experiments. Scale bar = 50 μm. Statistical significance was calculated using the one-way ANOVA with Tukey’s multiple comparison.*p < 0.05; **p < 0.01; ***p < 0.001 and ****p < 0.0001

EMT is a process mandatory for the local and distant progression of many malignant tumours, in which MMPs play a significant role. To verify the activation of these enzymes, control HEK293, DDB2^Wt^ and DDB2^PCNA−^ culture media were collected at different recovery time after UV irradiation. Representative images of the results obtained by time course experiments are shown in Fig. 3A. The white bands at 84 kDa confirmed the activity of MMP-9, as quantified in Fig. 3B. Until 48 h after UV-induced DNA damage, the MMP-9 activity was quite similar in all the three cell lines. In DDB2^PCNA−^ cells, it started to increase time-dependently from 72 h, and this trend was maintained until 168 h; at this time, MMP-9 activity was almost 2-fold higher compared to its unirradiated control (*p* < 0.0001), and significantly higher *versus* DDB2^Wt^ clone (*p* < 0.05 at 72 h; *p* < 0.01 at 96 and 168 h) considering the last three time points. Conversely, no significant change in the amount of MMP-2 as detected by bands at 62 kDa, was observed (Fig. S4).

### UV-irradiated DDB2^PCNA−^ cells show the migration and invasion abilities

We have previously demonstrated by Wound healing assay that irradiated HEK293 DDB2^Wt^ and, even more the mutant stable clone, acquire both proliferation and migration advantages [19].

To further investigate whether the DDB2^PCNA−^ protein influences cell migration, after UV-C damage, a Boyden chamber assay was performed. All the unirradiated cells of the three cell clones were able to migrate in the same way (data not shown) in the presence of FBS as a chemoattractant *stimulus*. Whereas, the irradiated DDB2^PCNA−^ clone acquired the best motility as demonstrated by an increased number of migrated cells (Fig. 3C and D). Interestingly, these cells formed cluster of cells, as compared to control cell line or DDB2^Wt^ clone (Fig. 3C).

Similar results were obtained using Boyden chamber assay in which the filter was conditioned with Matrigel, a useful substrate to test cell invasion ability. In the absence of chemoattractant stimulus (0% FBS), a very low number of invading cells was observed (data not reported). Instead, as shown in Fig. 3E and F, cells expressing DDB2^PCNA−^ protein are able to cross the filter in the presence of 10% FBS. The number of cells skilled to invade in the mutant sample is 4-fold higher than that of the two other cell clones; in addition, no significant difference was observed between control and DDB2^Wt^ samples, suggesting that the invasion ability is not influenced by DDB2^Wt^ overexpressed protein (Fig. 3F).

Taken together, these results demonstrate that the loss of DDB2-PCNA interaction determines an increased capability of the cells to migrate and to invade.

### Alterations of EMT-TFs levels in DDB2^PCNA−^ cells

Starting to analyse the molecular mechanism underlying EMT induction by UV irradiation in DDB2^PCNA−^ clone, the expression level of a restricted number of transcription factors, which are known to orchestrate EMT (EMT-TFs), such as the three NF-kB, SNAI1, and ZEB1 was investigated.

**Fig. 4:**
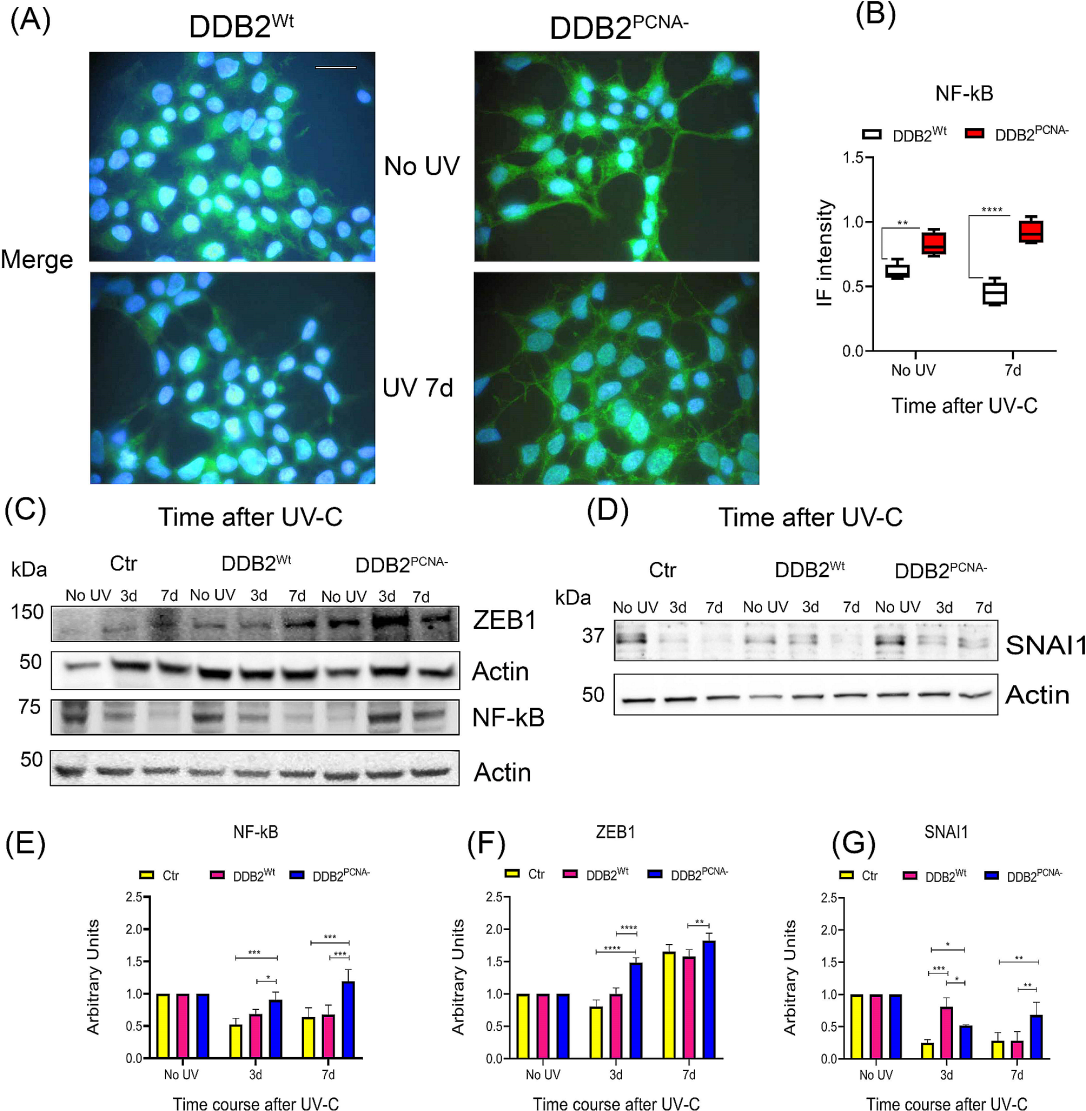
EMT-TFs levels in UV-irradiated HEK293 stable clones. Immunofluorescence analysis of NF-kB cellular distribution (**A**) and the quantification analysis performed by Image J software analysis (**B**) are reported. Irradiated cells collected at different time are used for Western blot analysis, as reported in Material and Methods section. Protein levels were normalized to actin values through densitometric analysis. Merged images of NF-kB (green) immunofluorescence and Hoechst staining (blue) are shown. In (**C**, **E**, and **F**) results from NF-kB and ZEB1; in (**D** and **G**) data from SNAI1 are shown. Data are the mean ± S.D. from at least three independent experiments. Scale bar = 50 μm. Statistical significance was calculated using the one-way ANOVA with Tukey’s multiple comparison.*p < 0.05; **p < 0.01; ***p < 0.001 and ****p < 0.0001

In the literature is reported that when NF-kB is localized in both nucleus and cytoplasm compartments is functionally active, while when it is present only in the cytoplasm it is inactive [23]. As shown in Fig. 4A and in Fig. S5, NF-kB is present in both in DDB2^Wt^ and DDB2^PCNA−^ cells. To evaluate the total amount of this protein in the cell, 7 days after UV-damage, we applied ImageJ software calculating the fluorescence present in whole cells. The results obtained confirm a higher fluorescence signals in HEK293 expressing DDB2^PCNA−^ protein than DDB2^Wt^ one, especially evident 7d after DNA damage induction (Fig. 4B). Western blot analysis evidenced a reduction of about 40% in protein level both in the control and DDB2^Wt^ samples at 3d and 7d after UV irradiation, as compared to the relative unirradiated sample. On the contrary, the NF-kB protein level measured in DDB2 mutant cells is not significantly modified respect to unirradiated cells (Fig. 4C and E). NF-kB activation induces transcription of downstream genes involved in principal hallmarks of cancer; among of these, there are SNAI1 and ZEB1 that play a key role in EMT process. The analysis of the SNAI1 protein level confirmed that at 7 days after UV irradiation, the cells expressing DDB2^PCNA−^ protein show a higher-level respect to that expressing DDB2^Wt^ or control that show a similar amount of the protein (Fig. 4D and G).

Finally, we demonstrated that ZEB1 protein level is significantly increased at 3 days after UV-damage and its amount remains stable until the end of the time-course (Fig. 4C and F). This result is crucial given that ZEB1 is a transcription factor promoting tumour invasion and metastasis by inducing EMT.

### DDB2 interacts with NF-kB and their association is increased in mutant cells

**Fig. 5:**
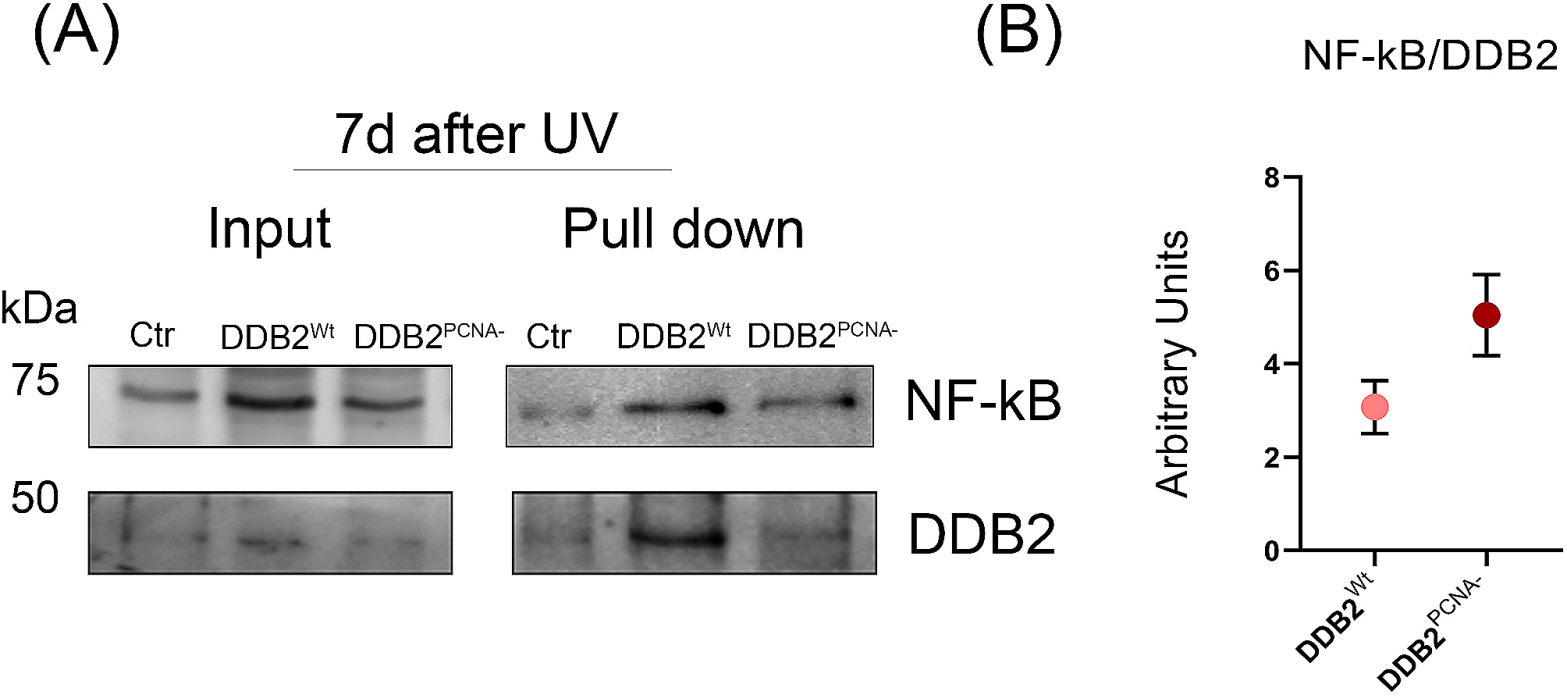
DDB2 and NF-kB protein interaction. Western blot analysis from pull-down samples obtained 7 d after UV-irradiation of the cells. In (**A**) total protein (Input) and pull-down samples from control HEK293 (Ctr), and the two stable clones DDB2^Wt^ or DDB2^PCNA−^ expressing His-tagged proteins. Samples are prepared as reported in Materials and methods section. The uncropped original blots are shown in the supplementary file WB. In (**B**), the related data from densitometric analysis of NF-kB/DDB2 ratio of DDB2^Wt^ or DDB2^PCNA−^ stable clones

To investigate a possible DDB2 and NF-kB protein interaction a pull-down assay was carried out. In Fig. 5A, a representative image of the pull-down experiment was shown in which both DDB2^Wt^ and DDB2^PCNA−^ are able to interact with the NF-kB protein; this interaction appears stronger in the presence of DDB2 mutant protein than that of the *wild-type* one. In fact, the quantification analysis, reported in Fig. 5B, highlights this novel result supporting this association between DDB2^PCNA−^ and NF-kB proteins.

### Modifications of DNA replication protein levels in irradiated DDB2^PCNA−^ cells

We have previously demonstrated that cells expressing DDB2^PCNA−^ protein show alterations in cell cycle progression [20] acquiring proliferative advantage after DNA damage [19].

**Fig. 6:**
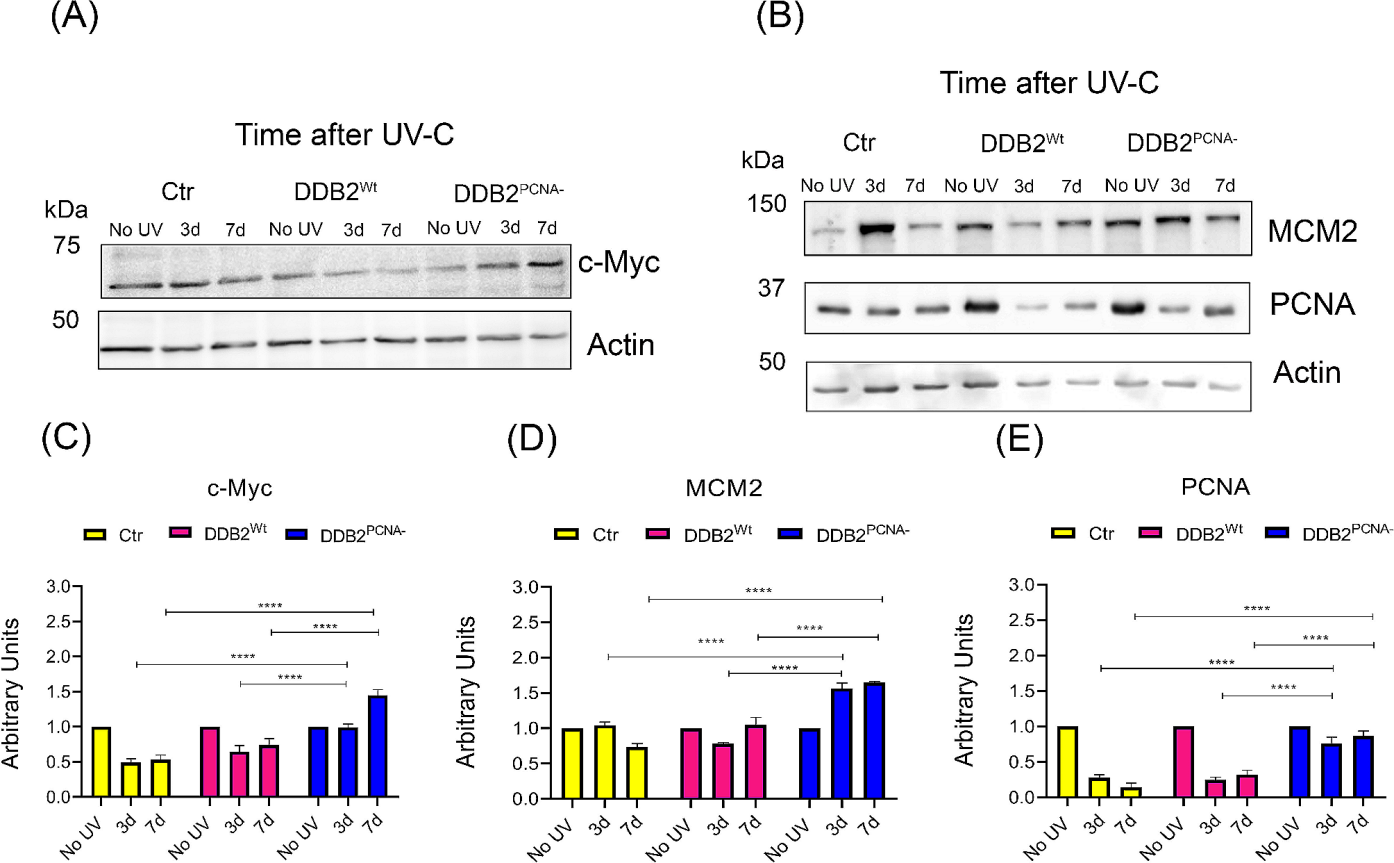
DNA replication protein levels in UV-irradiated HEK293 stable clones. Representative images of Western blot analysis and statistical analysis of c-Myc (**A** and **C**), MCM2 (**B** and **D**) and PCNA (**B** and **E**). The uncropped original blots are shown in the supplementary file WB. Cells were irradiated and collected as reported in Material and Method section. Protein levels were normalized to actin values through densitometric analysis. Data are the mean ± S.D. from at least three independent experiments. Statistical significance was calculated using the one-way ANOVA with Tukey’s multiple comparison.****p < 0.0001

Here, we investigate the protein level of c-Myc, an oncogene that influences DNA replication and regulates the transcription of EMT-TFs. In DNA replication field, it stimulates the transcription of factors involved in late M/early G1 phase such as MCMs proteins that are involved in the assembly of pre-replicative complex; among these, we evaluate MCM2 protein. We also study the PCNA protein level that is a key factor in S-phase of cell cycle progression. In Fig. 6, we reported representative images (A and B) and graphical summary of collected data (C, D, E) that demonstrate similar trends in protein levels of c-Myc, MCM2 and PCNA in control cells and those expressing DDB2 in the wild-type form after UV-C irradiation. Instead, in cells expressing DDB2 unable to interact with PCNA, the protein levels of all three factors considered are higher than the other two clones; these data seem to confirm that mutant cells do not block their proliferation in the presence of DNA damage.

### UV-irradiated DDB2^PCNA−^ cells show higher HPRT levels and reduced clonogenic efficiency after 6-thioguanine and cisplatin drugs treatment

To further validate the acquisition of malignant phenotype by DDB2 mutant cells, we assessed the level of HPRT, an enzyme regulating nucleotide synthesis whose overexpression and activity contribute to tumour progression [24, 25]. In fact, this enzyme plays an important role in influencing the cell cycle progression through modulation of guanine and inosine production in the salvage pathways [26]. Moreover, its use as diagnostic biomarker has been suggested in cancer field [27].

**Fig. 7:**
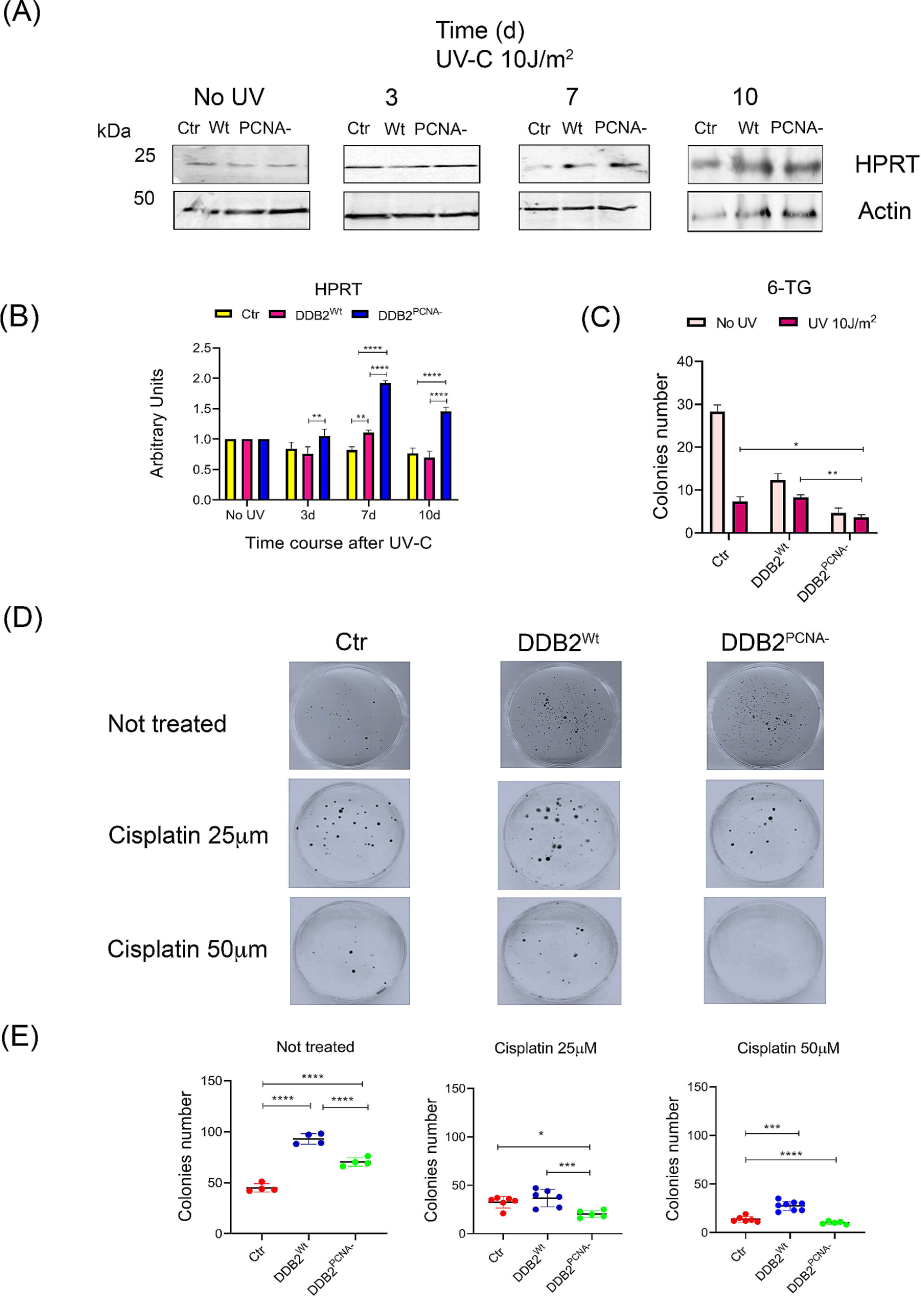
Clonogenic efficiency after drug treatments. DDB2^PCNA−^ cells showed an increase in HPRT level and activity. In (**A**) representative images from cropped blots, as indicated in material and methods, are presented. The uncropped original blots are shown in the supplementary file WB. HEK293 control cells (Ctr) and DDB2^Wt^ or DDB2^PCNA−^ clones are irradiated and harvested until to 10 days after UV irradiation (see Materials and Methods). Results from densitometric analysis are reported in (**B**) and data from clonogenic assay in irradiated and not irradiated cells treated with 6-TG cytotoxicity in (**C**). In (**D**), the colonies grown after Cisplatin treatment are shown (for details see Material and methods section) and the statistical analysis is reported (**E**). Data are the mean ± S.D. from at least three independent experiments. Statistical significance was calculated using the one-way ANOVA with Tukey’s multiple comparison. *p < 0.05; **p < 0.01, ***p < 0.001 and ****p < 0.0001

Figure 7 shows that HPRT expression appears to increase in mutant cells both at 7 d and 10 d after UV-damage, reaching significantly higher value than that of the corresponding unirradiated sample. In control and in cells expressing DDB2^Wt^ protein, no significant variations were observed except for the strong reduction in DDB2^Wt^ clone at 10 d (Fig. 7A and B**).**

The anticancer activity of 6-thioguanine (6-TG) drug is dependent on HPRT enzyme ability to insert in DNA this base-analogous [27]. In our experimental model, in which we observed an increased amount of this protein, HEK293 clones were exposed to the toxic nucleotide analogue and we performed a clonogenic assay. The results indicated that the 6-TG-treated mutant clone is not able to form colonies, both before and after UV irradiation while in both UV-irradiated control and DDB2^Wt^ cells, the number of colonies was higher than that of the mutant clone (Fig. 7C). These data suggest that cells expressing DDB2^PCNA−^ protein incorporate 6-TG faster than the other cells confirming an increased proliferation as previously published [19].

In addition, we performed a clonogenic assay using non-irradiated cells treated with Cisplatin, a chemotherapic drug used to treat different human cancers. As reported in Fig. 7D and E, we demonstrated that the mutant clone is more sensitive against this drug than the cells expressing DDB2^Wt^ protein. This result was confirmed using two different concentrations of the drug and a correlation dose-response was highlighted.

## Discussion

While DDB2 is considered a suppressor of the EMT process in colon cancer cells [28–30], recent lines of evidence have indicated that this protein may induce EMT in other types of cancer [6–8]. In this study we have investigated the occurrence of EMT in HEK293 cells expressing DDB2^PCNA−^, because these cells were previously shown to favour cell proliferation and motility. In particular, we have analysed the appearance of EMT markers in cells expressing DDB2^Wt^*versus* those expressing DDB2^PCNA−^ protein, after inducing DNA damage with UV irradiation. The mutant cell clone showed a reduced expression of E-Cadherin and an increased activity of MMP-9 compared to the unirradiated and UV-irradiated samples of both non-transfected control and DDB2^Wt^ clones. These data support the hypothesis that DDB2^PCNA−^ cells were able to move towards a mesenchymal state, acquiring mobility and capacity to migrate and invade. In addition, a statistically significant increase in Vimentin levels was found in DDB2^PCNA−^ clone, although the absolute difference in comparison with the wild-type clone was relatively small. We speculate that in the mutant cells there might be in progress a dynamic EMT (p-EMT) state, thereby suggesting that the DDB2^PCNA−^ triggers the onset of mesenchymal cell phenotype with respect to the Wt form. In fact, it has been reported that the change from epithelial-to-mesenchymal state is very often incomplete, resulting in cells maintaining both E/M characteristics [31], yet able to participate in cancer initiation and metastasis [3, 32, 33]. The biochemical changes, such as a reduced intercellular junction (E-Cadherin) and an increased matrix remodelling (Metalloproteases), observed in the mutant clone confirm the activation of EMT process, also supported by the presence of morphological changes, such as an elongated mesenchymal shape of the DDB2^PCNA−^ cells. Moreover, these results are confirmed in HeLa cells expressing DDB2 mutant protein as there is an increase in the Vimentin expression, a known marker of mesenchymal phenotype.

These morphological observations are in agreement with previous findings showing that DDB2 is involved in modulating nanomechanical properties and stiffness, associated with changes in the cortical actin–cytoskeleton organization and loss of adhesion capacity [16]. By using both iCELLigence biosensor technology and Boyden chamber experiments, we confirmed the greater ability of DDB2^PCNA−^ cells to move, migrate and invade, all of which are linked to EMT activation [34]. In concomitance, we have observed a significant increase in the levels of NF-kB, which is notably involved in tumour promotion, EMT, metastasis formation and cell proliferation, in cells expressing DDB2^PCNA−^, as compared with DDB2^Wt^ cells. Consistent with these results, the expression of crucial downstream genes such as MMPs, Snail and Zeb family genes, as well as of c-Myc and NF-kB [35–44], was also increased in DDB2^PCNA−^ cells, as compared with the Wt clone. Altogether, these findings support the idea that the mutant DDB2 form may promote the appearance of cell features associated with a malignant phenotype, showing increased protein levels of c-Myc, ZEB1 and NF-kB.

Interestingly, an interaction between DDB2 and NF-kB, was found by pull-down in both cell clones, yet it was more evident in cells expressing DDB2^PCNA−^. This novel result open to an interesting cross-talk between DDB2 and NF-kB, and suggests that the absence of the interaction with PCNA may influence DDB2 functions associated with the expression of the above TFs. However, further work is necessary to understand the mechanism underlying these functions.

The regulation of EMT process also influences negatively the chemotherapic response [13, 45]. Consistent with our previous results, highlighting that cells expressing DDB2 mutant protein acquire a proliferative advantage [19], we have now demonstrated a reduced clonogenic efficiency in the presence of 6-TG and an increased HPRT protein level after cells being damaged by UV. 6-TG is an anticancer drug producing a cytotoxic metabolite leading to the p53-dependent intrinsic apoptosis [46]. Our results indicate that cells expressing DDB2^PCNA−^ protein are more susceptible to 6-TG treatment in agreement with those obtained from the HPRT expression analysis. HPRT is a salvage pathway enzyme involved in synthesis of nucleotide recycling parts from old nucleotides. Consequently, in the DDB2^PCNA−^ cells, both the higher proliferation rate and increased HPRT level determine a greater DNA replication and, therefore, an incorporation of the drug 6-TG which leads to cell death. Interestingly, *hprt* gene expression is increased in different tumours [47–49]. Considering the significant increase of its expression in the presence of DDB2^PCNA−^ protein, we speculate that HPRT may contribute to the transition from transformed phenotype towards cancer phenotype. It has been reported that tumours with DDB2 deficiency should be more sensitive to DNA-damaging treatments, such as platinum products [50]. In fact, cells overexpressing DDB2^Wt^ protein were more resistant to cisplatin. However, we have previously demonstrated that cells expressing DDB2^PCNA−^ protein show a delay in DNA damage removal, acquiring a proliferative advantage, i.e., resistance to UV damage [19]. In contrast, the mutant clone was found to be more sensitive to cisplatin. A possible explanation could be that this drug induces DNA lesions (cross-links and double-strand breaks) that, in the presence of a defective NER, activate a molecular transcriptional program resulting in cell cycle arrest or apoptosis [51, 52].

In conclusion, although the direct DDB2-PCNA interaction could be involved in preventing the onset of aggressive cell phenotype after UV-induced damage, we cannot exclude a DDB2 role in other cellular pathways. The new evidence that DDB2 interacts with NF-kB remarkably supports the hypothesis that DDB2 may be involved in early transcriptional events occurring before metastases formation thus inhibiting cancer progression.

### Electronic supplementary material

Below is the link to the electronic supplementary material.

Supplementary Material 1

Supplementary Material 2

## Data Availability

The datasets used and/or analysed during the current study are available from the corresponding author on reasonable request.
