# Supplementary material for: Epithelial-to-mesenchymal transition and NF-kB pathways are promoted by a mutant form of DDB2, unable to bind PCNA, in UV-damaged human cells
Source: BMC Cancer. 2024 May 21;24:616. doi: 10.1186/s12885-024-12368-6 (PMC11110260; doi:10.1186/s12885-024-12368-6)
Supplement: Supplementary file 1 — Supplementary Material 1 [file 12885_2024_12368_MOESM1_ESM.docx]

**Supplementary figure legends**

**
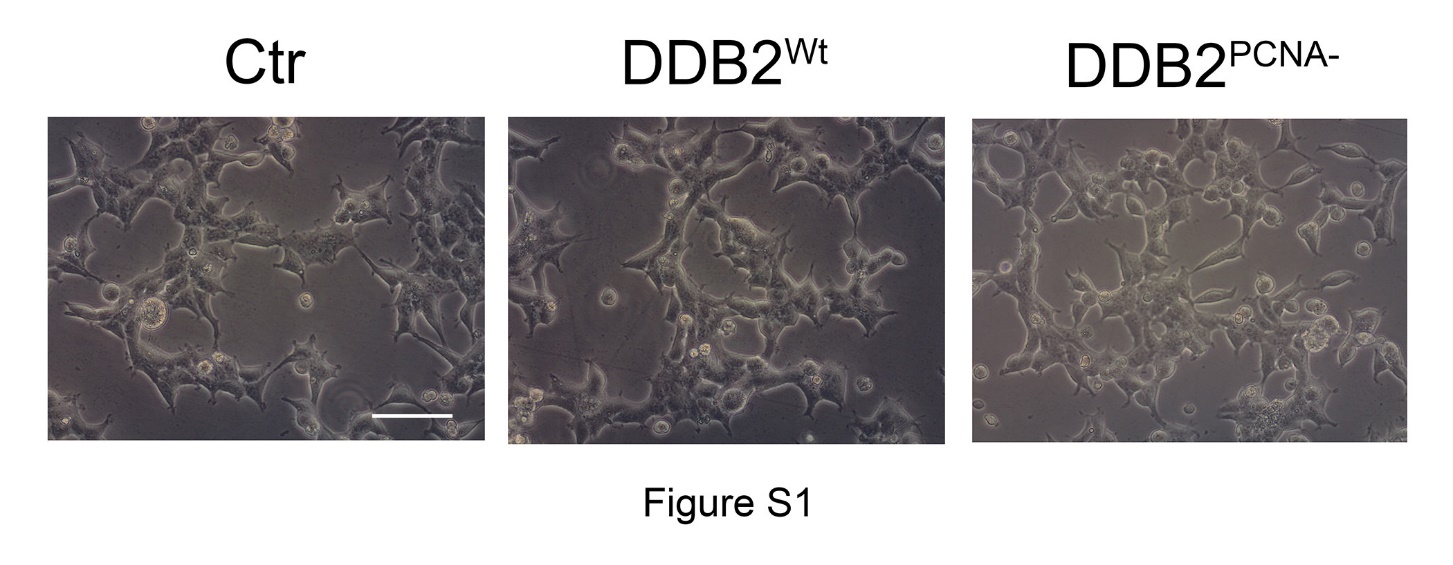
**

**Figure S1 -**  Representative images of morphological phenotypes of unirradiated control (Ctr), DDB2^Wt^ or DDB2^PCNA-^ clones (scale bar = 50 μm).


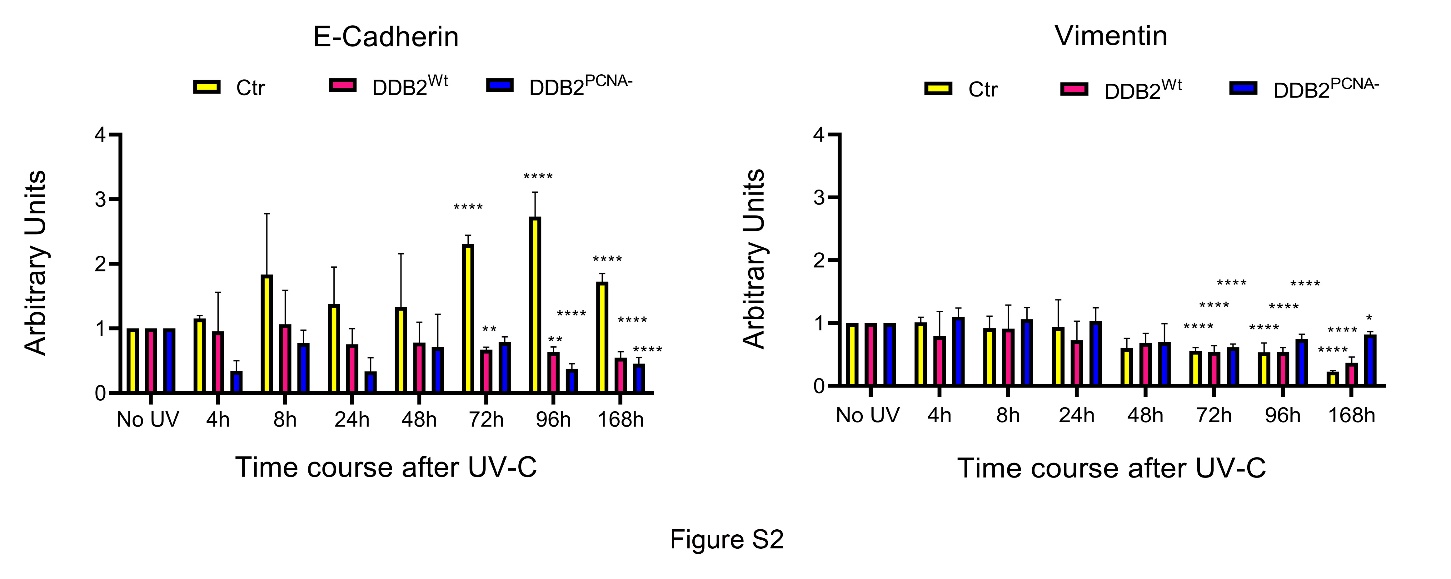


**Figure S2** - Complete time-course of E-Cadherin and Vimentin expression after UV damage. Data are the mean ± S.D. from at least three independent experiments. Statistical significance was calculated using the one-way ANOVA with Tukey’s multiple comparison; *p<0.05; **p<0.01; ***p<0.001 and ****p<0.0001. The statistical significance, calculated at different times after UV, is towards the unirradiated samples.


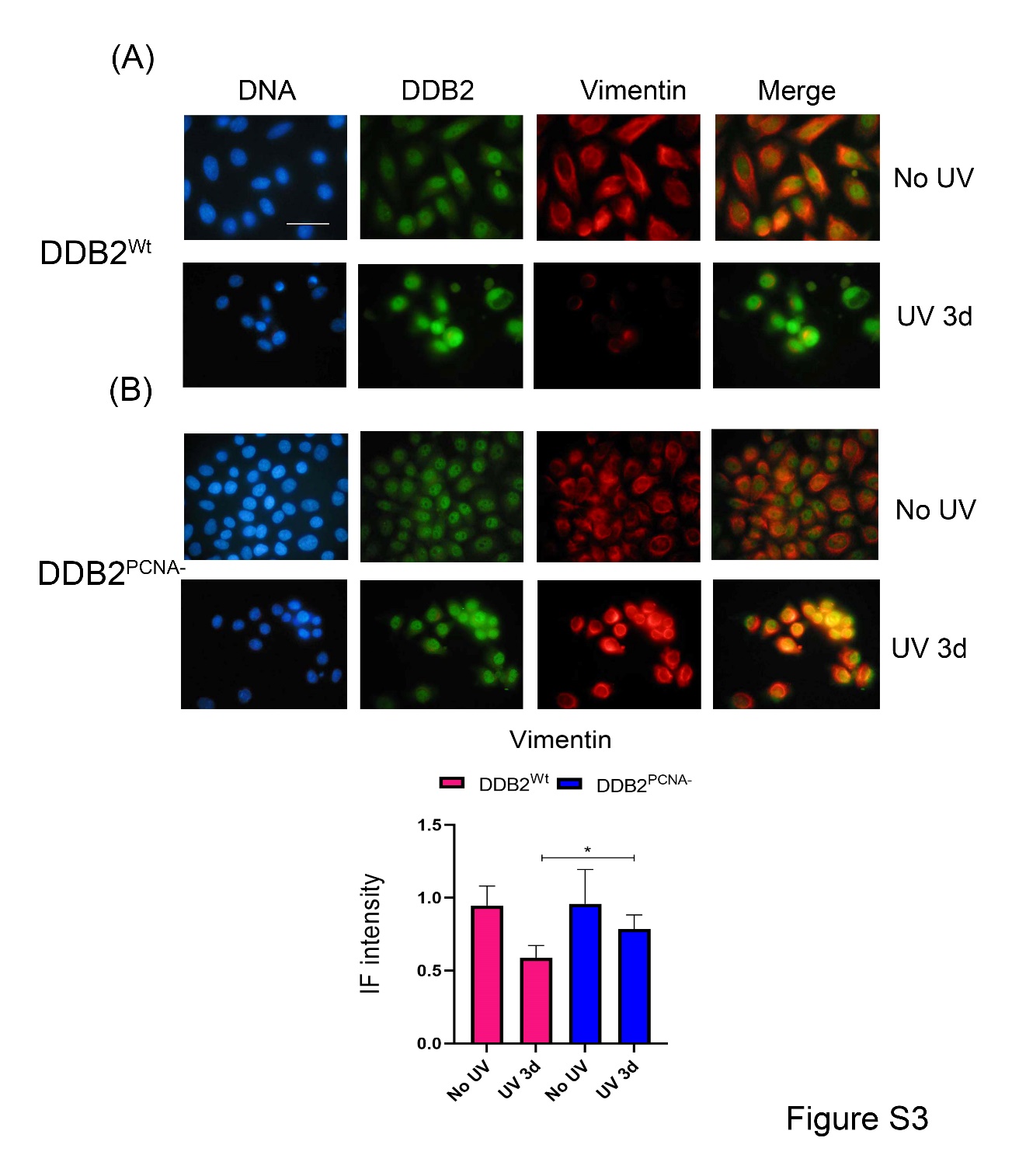


**Figure S3** - Immunofluorescence analysis of Vimentin expression. HeLa cells transiently transfected with DDB2^Wt^ or DDB2^PCNA-^ constructs were irradiated and stained in blue (DNA), green (DDB2) and red (Vimentin); (scale bar = 50 μm).


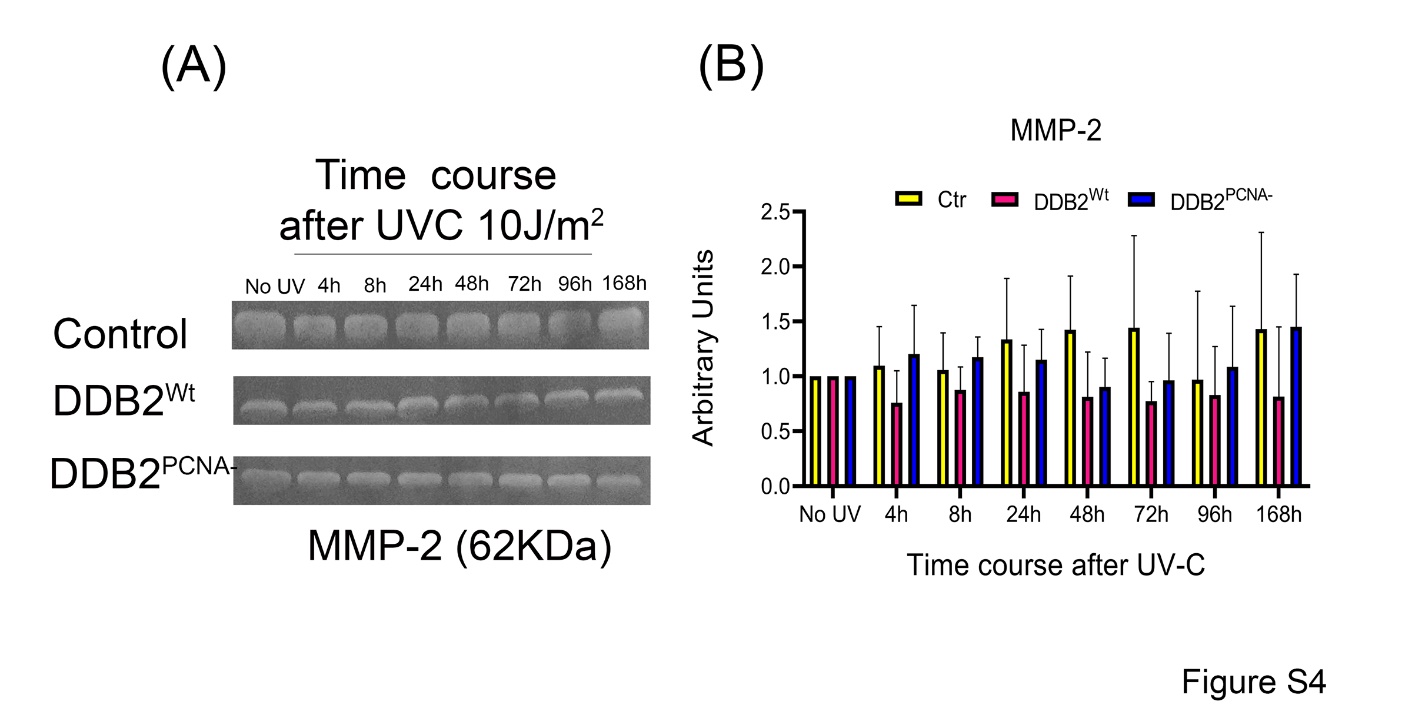


**Figure S4 -** Time course of MMP2 activity. Gel electrophoresis of MMP2 (A) and densitometric analysis of the obtained results (B).


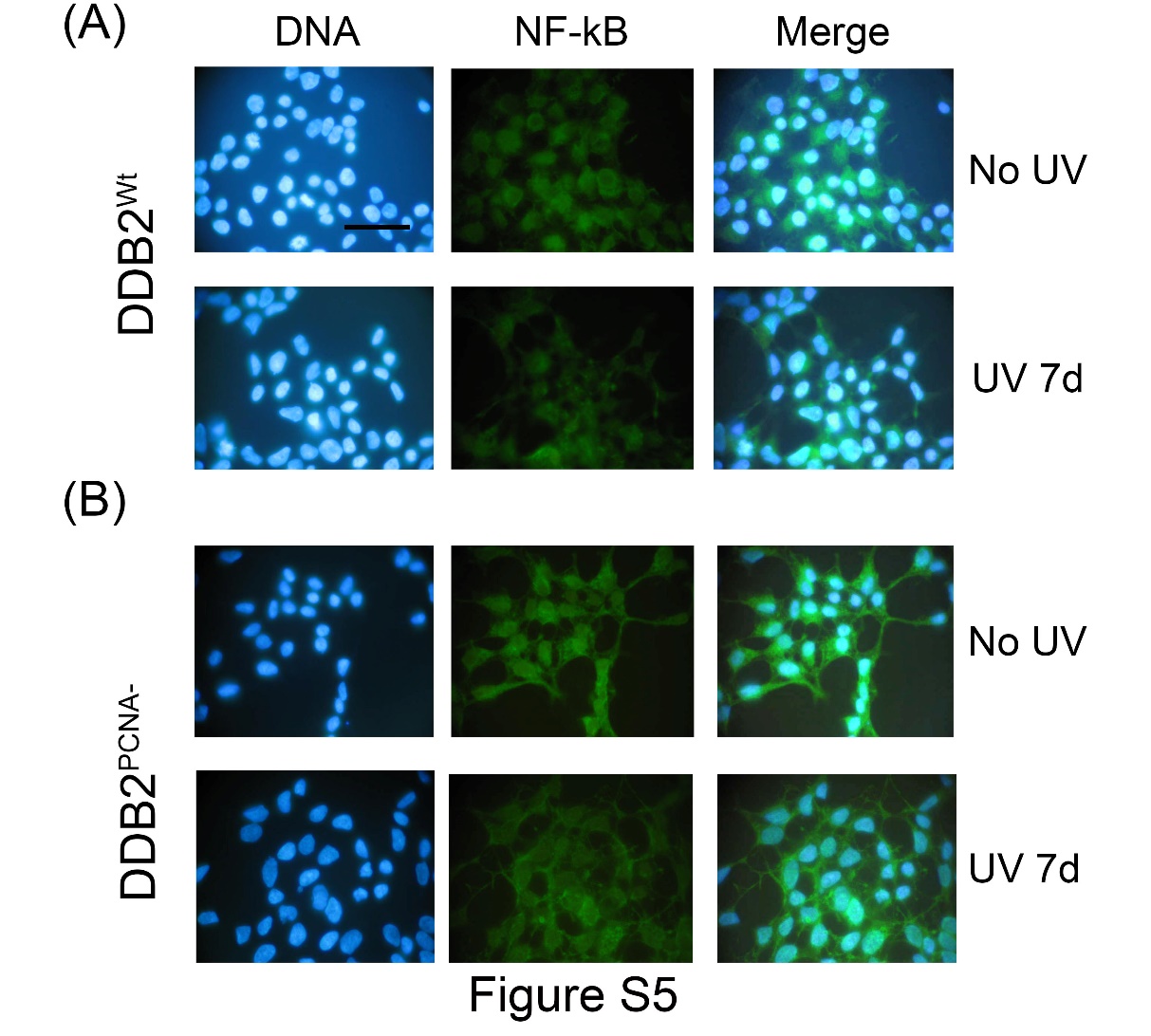


**Figure S5 -** Immunofluorescence analysis of NF-kB cellular distribution in untreated and UV-treated DDB2^Wt^ and DDB2^PCNA-^ clones. First panel DNA (blue, Hoechst staining), second panel NF-kB (green), third panel (merge) (scale bar = 50 μm).

**WB** – Original uncropped WB images of Figure 1 (1C and 1E), Figure 4 (4C and D), Figure 5 (5A), Figure 6 (6A and 6B), and figure 7 (7A).
