# Supplementary material for: Epithelial-to-mesenchymal transition and NF-kB pathways are promoted by a mutant form of DDB2, unable to bind PCNA, in UV-damaged human cells
Source: BMC Cancer. 2024 May 21;24:616. doi: 10.1186/s12885-024-12368-6 (PMC11110260; doi:10.1186/s12885-024-12368-6)

Fig 1C

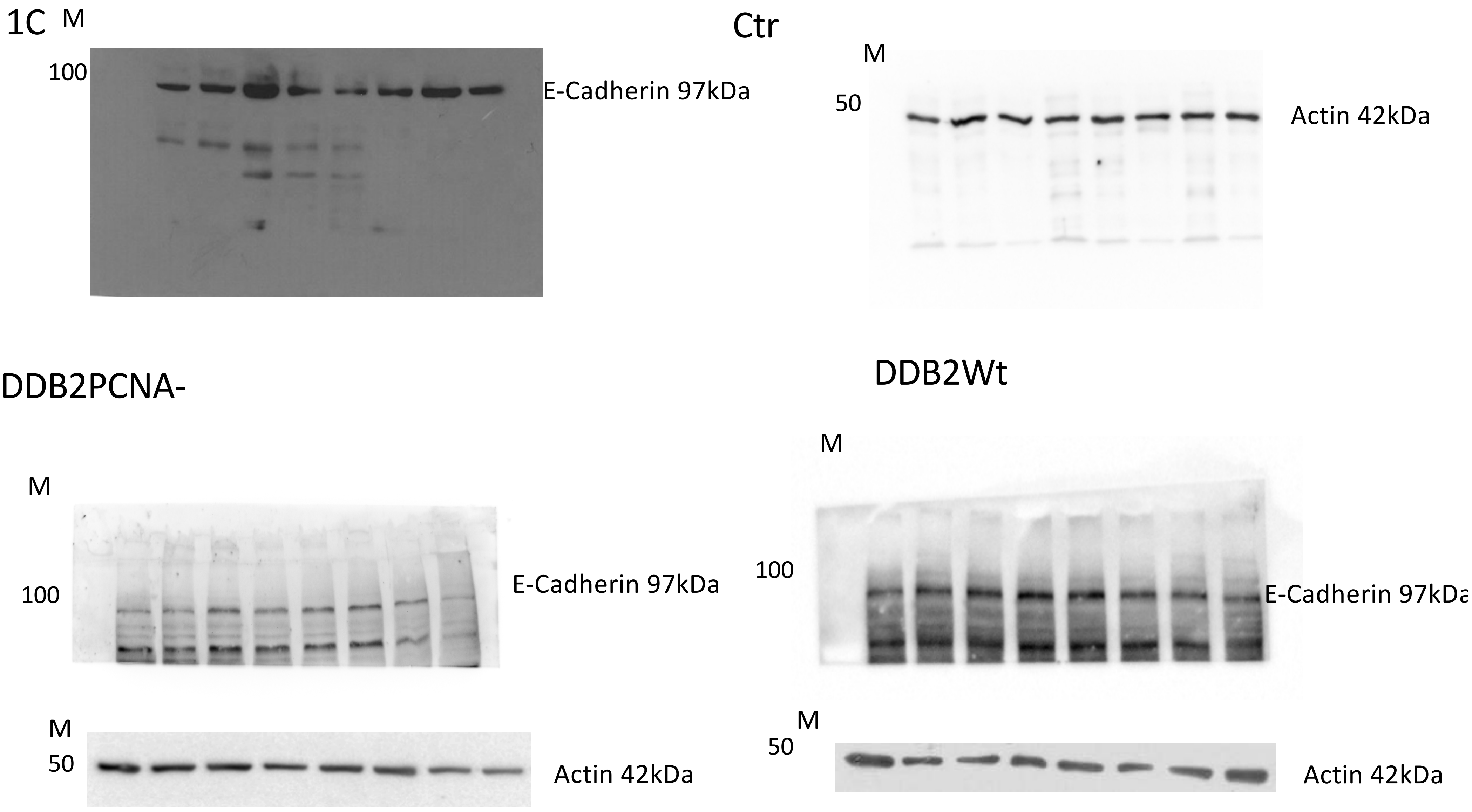
M
100
E-Cadherin 97kDa
Ctr
M
50
Actin 42kDa
DDB2PCNA-
M
100
E-Cadherin 97kDa
M
50
Actin 42kDa
DDB2Wt
M
100
E-Cadherin 97kDa
M
50
Actin 42kDa

Fig 1C

The same blots of actin for E-cadherin but more contrasted

DDB2PCNA-

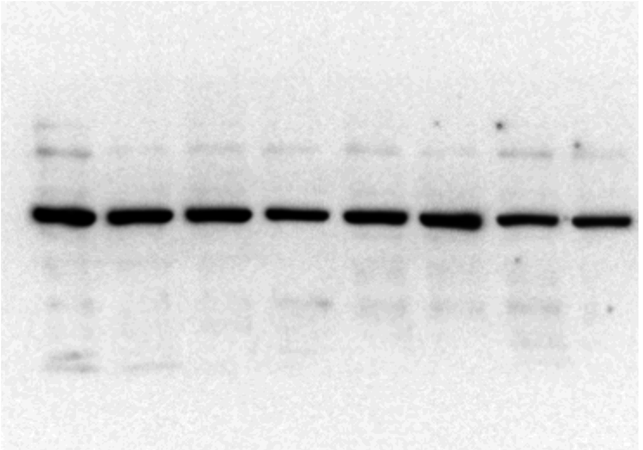

DDB2Wt

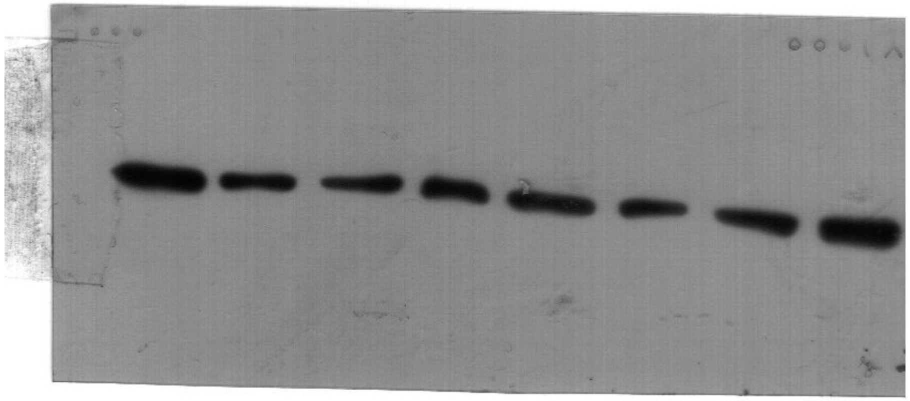

Fig 1E

CTR

M

75

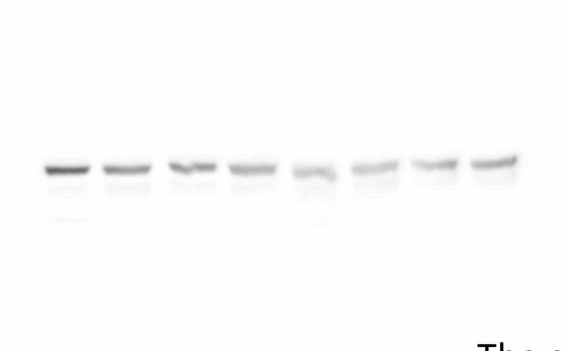

Vimentina 57 kDa

M

50

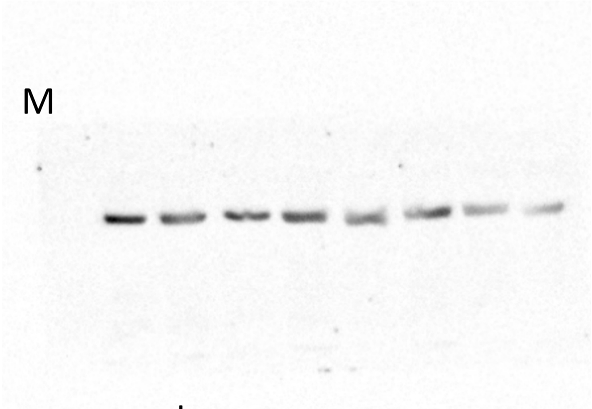

Actin 42 kDa

The same blots as above but more contrasted

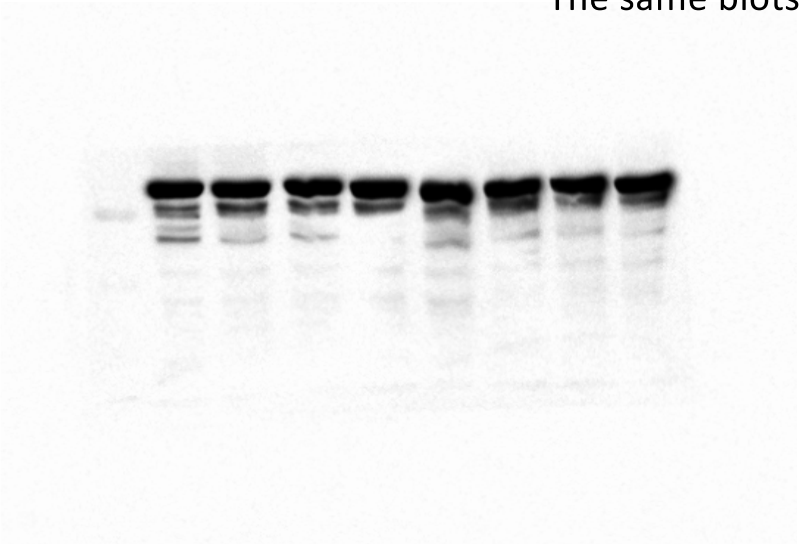

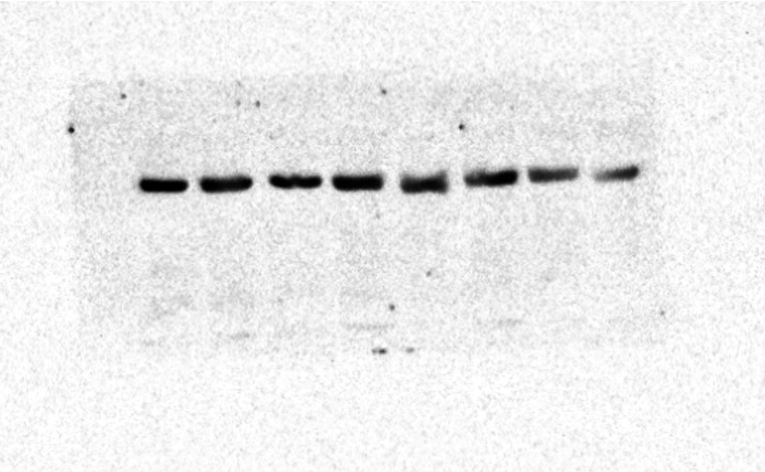

Fig 1E

DDB2WT

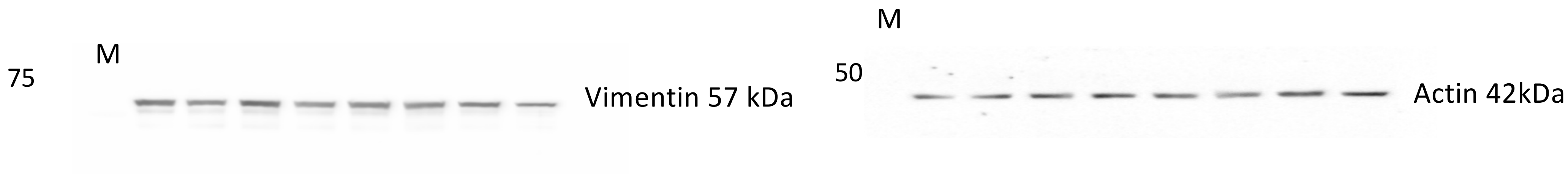

The same blots as above but more contrasted

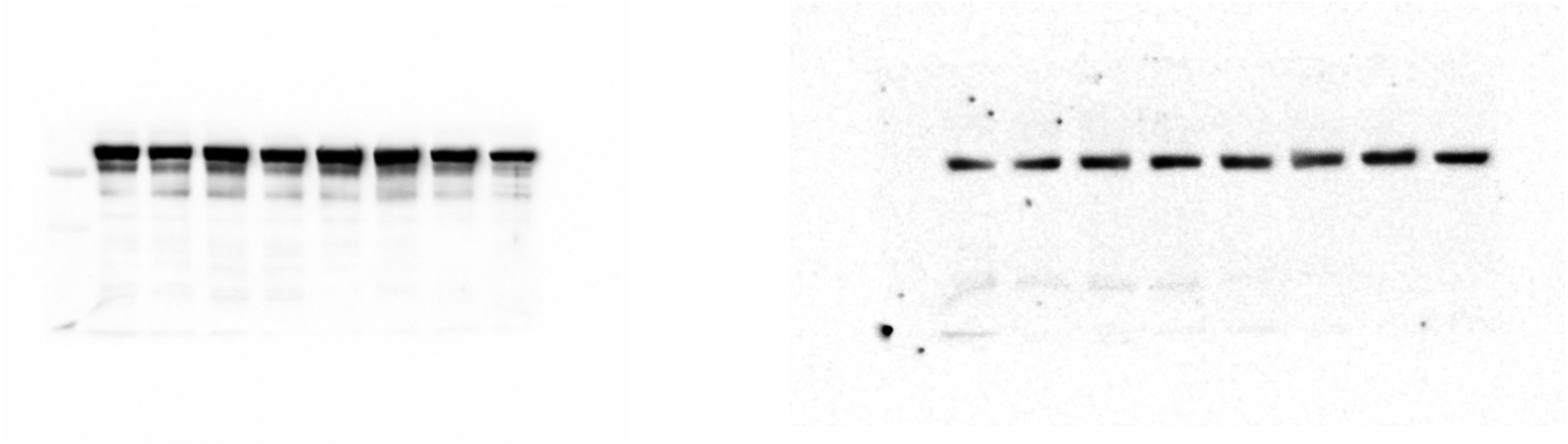

Fig 1E

DDB2PCNA-

M

75

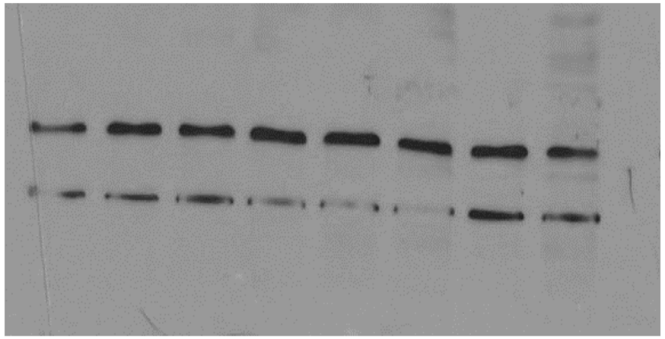

Vimentin 57 kDa

M

50

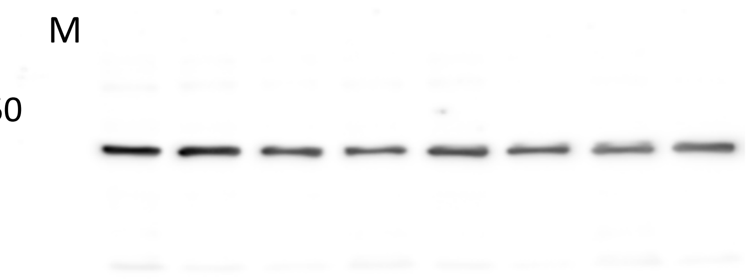

Actin 42kDa

The same blot as above but more contrasted

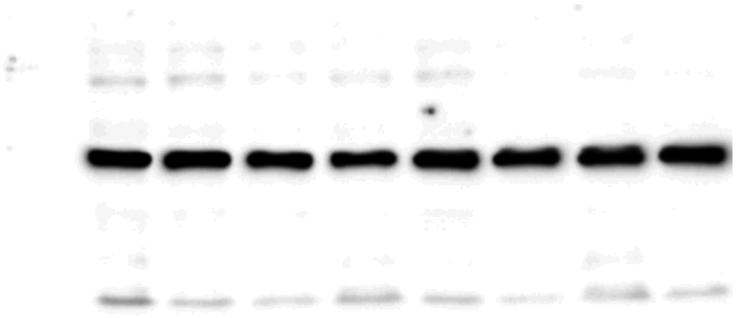

Fig 4D

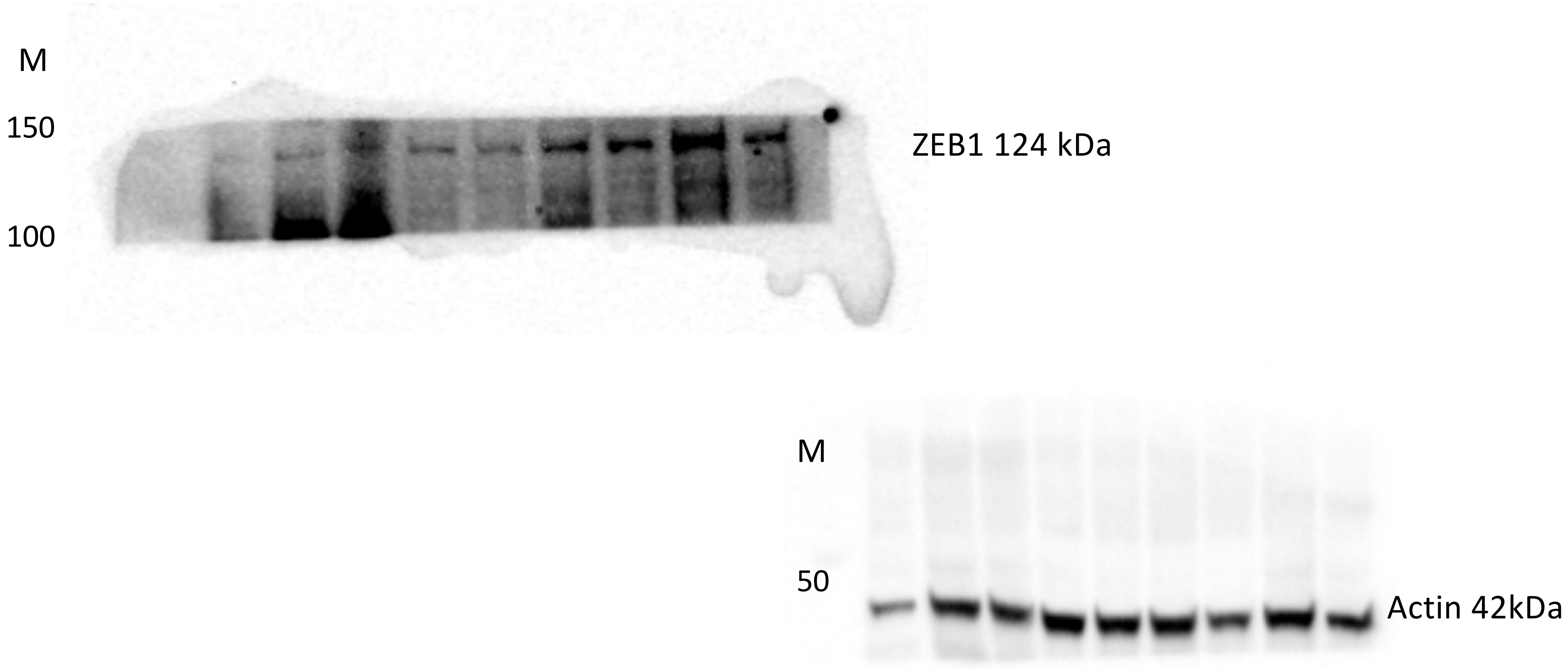

M

75

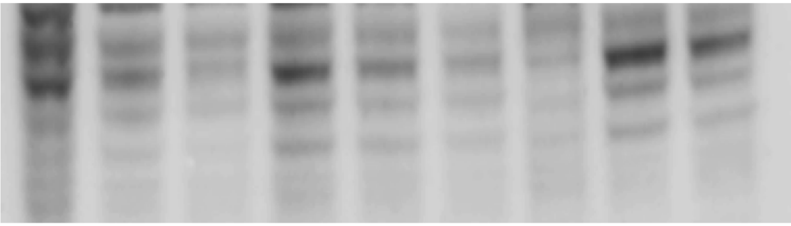

NF-kB 65 kDa

M

75

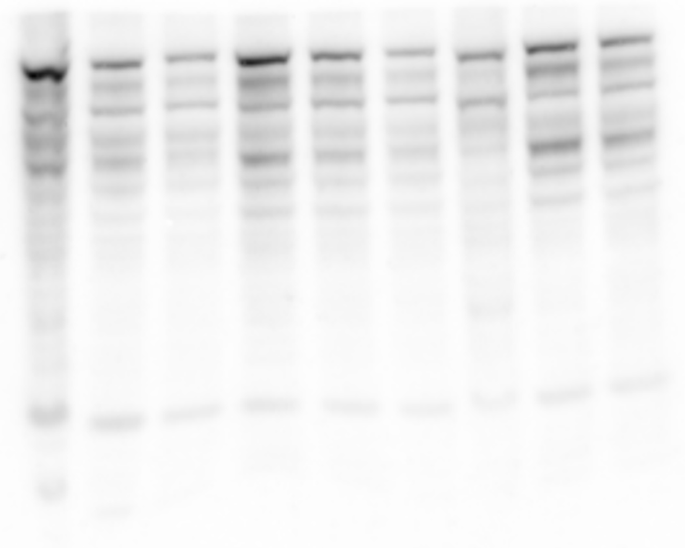

M

50

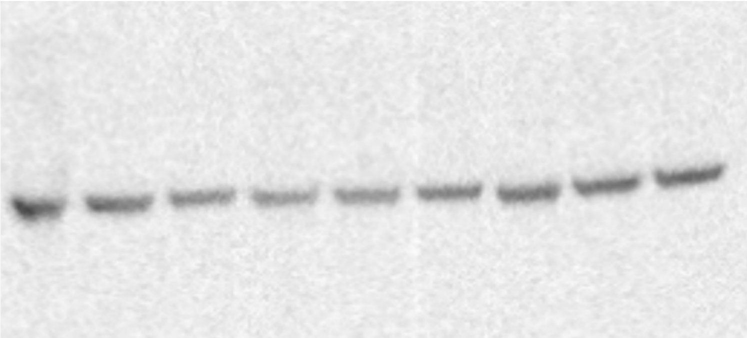

Actin 42kDa

Fig 4D

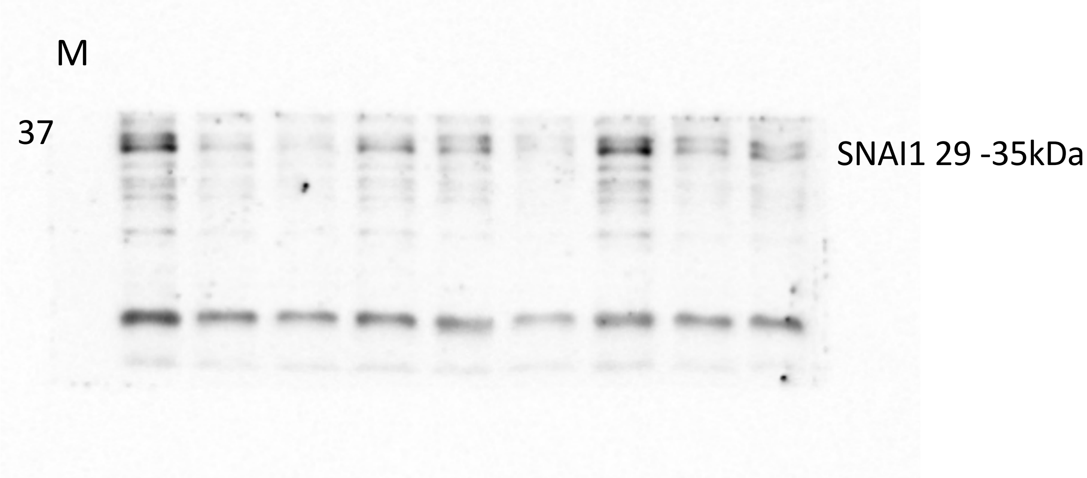

SNAI1 29 -35kDa

M

37

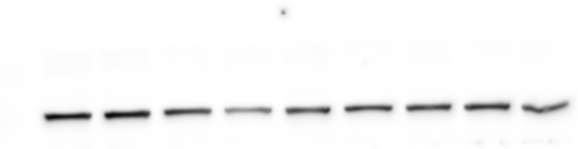

Actin 42kDa

The same blot above more contrasted

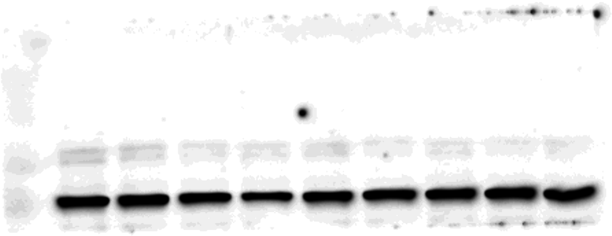

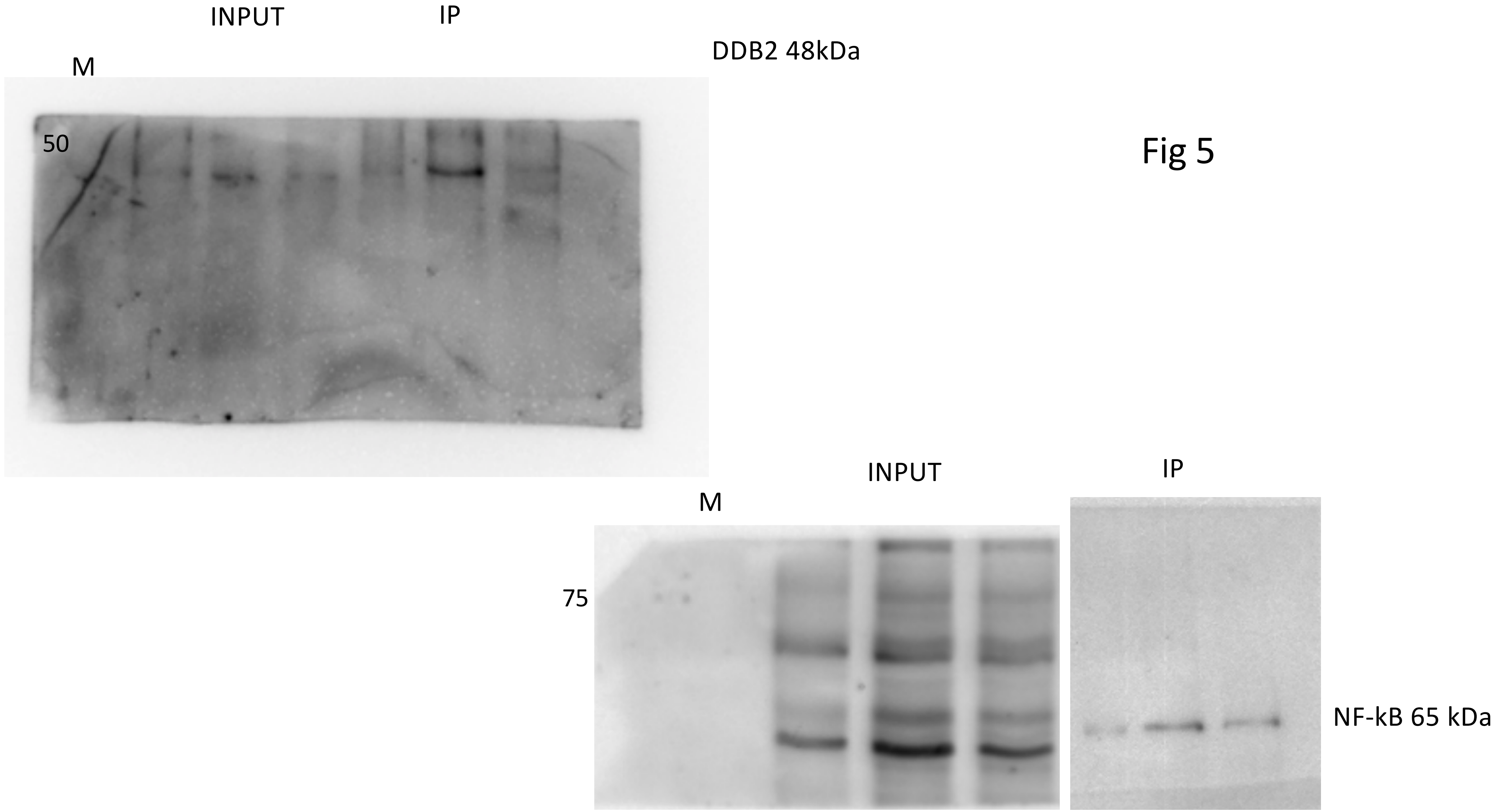

Fig 5

Fig 6A

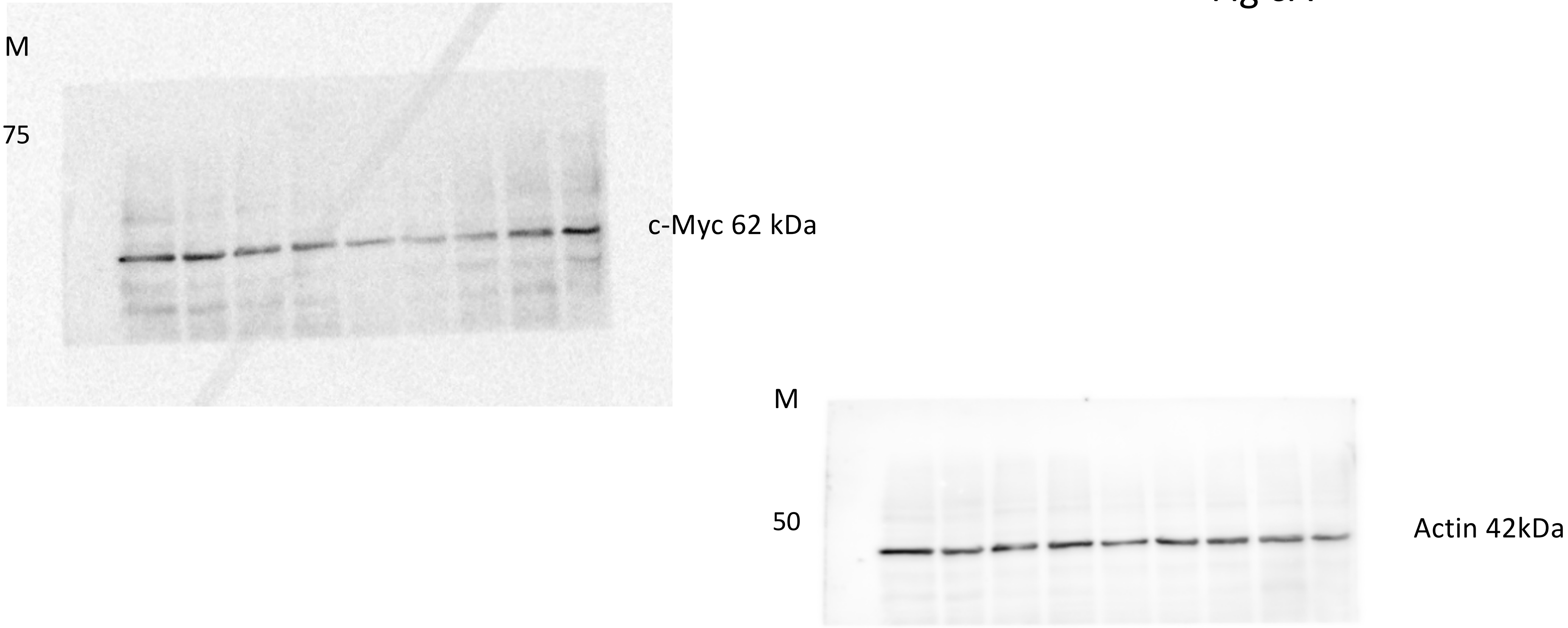

Fig 6B

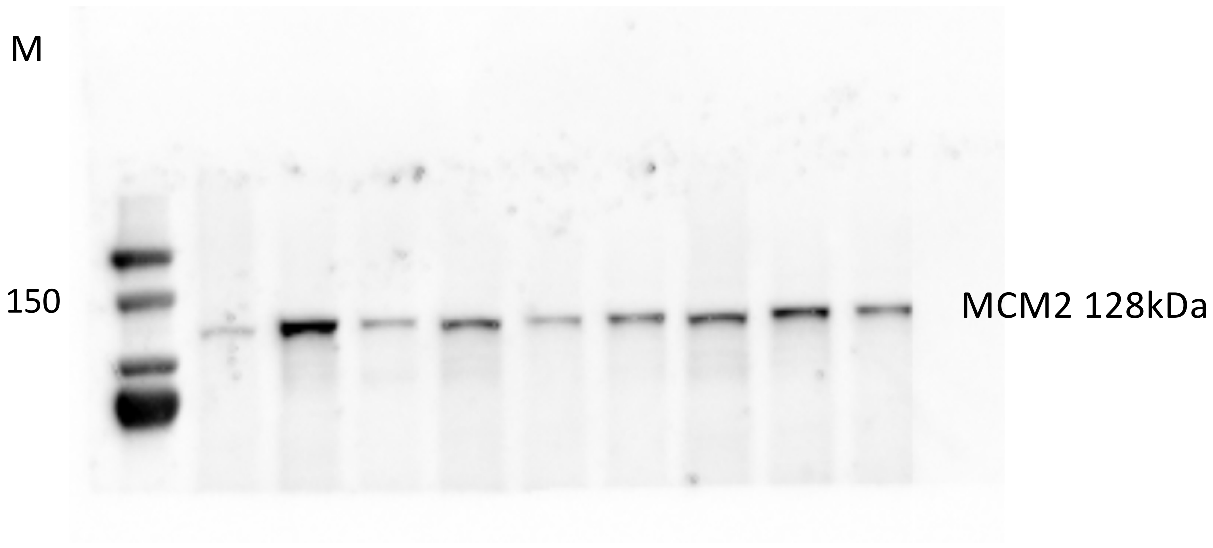

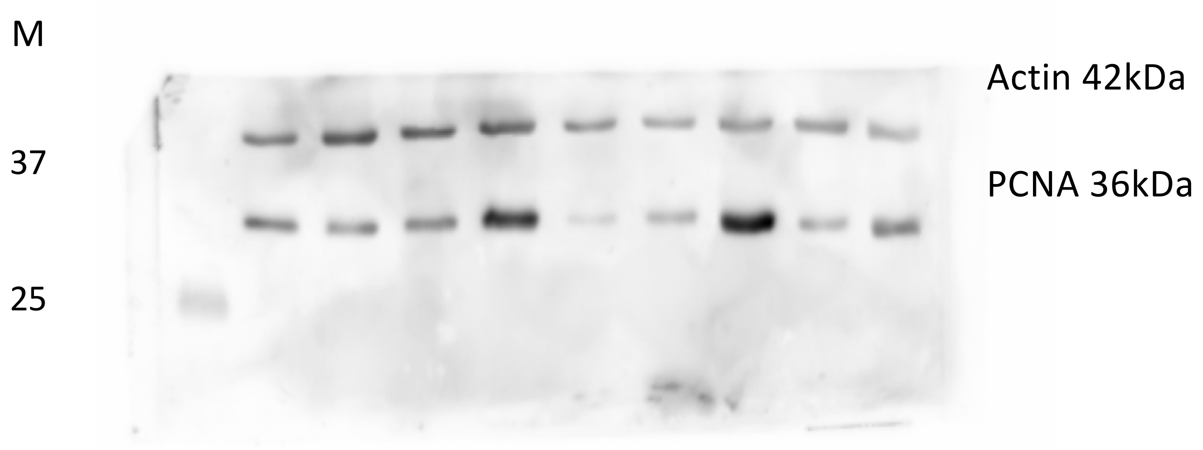

Fig 7

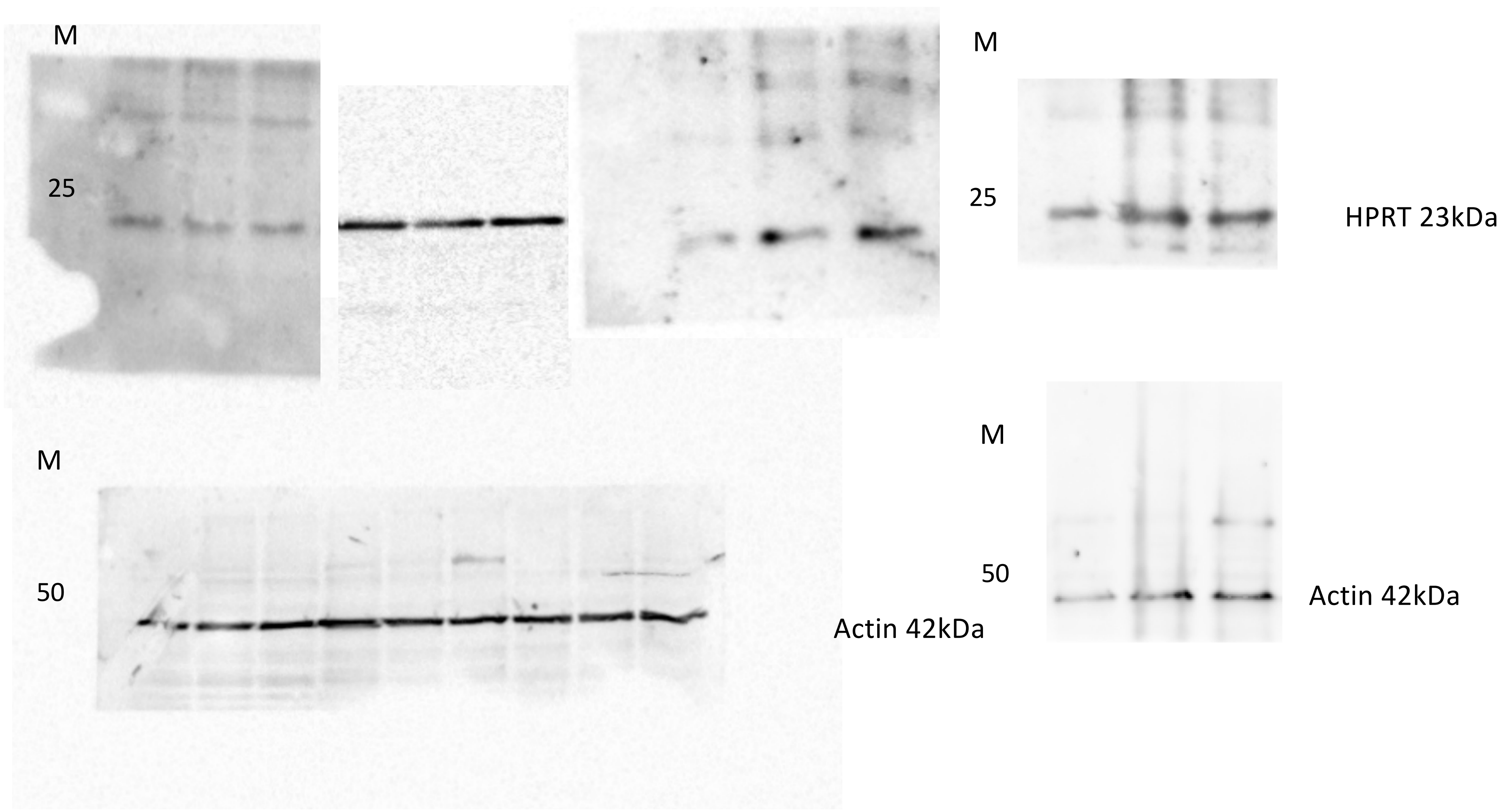

Supplement: Supplementary file 2 — Supplementary Material 2 [file 12885_2024_12368_MOESM2_ESM.pdf]
